# Gut Microbiota, Probiotics and Psychological States and Behaviors after Bariatric Surgery—A Systematic Review of Their Interrelation

**DOI:** 10.3390/nu12082396

**Published:** 2020-08-10

**Authors:** Jessica Cook, Christine Lehne, Alisa Weiland, Rami Archid, Yvonne Ritze, Kerstin Bauer, Stephan Zipfel, John Penders, Paul Enck, Isabelle Mack

**Affiliations:** 1 Department of Psychosomatic Medicine and Psychotherapy, University Medical Hospital, 72072 Tübingen, Germany; jessica.cook@med.uni-tuebingen.de (J.C.); ch-lehne@gmx.de (C.L.); alisa.weiland@med.uni-tuebingen.de (A.W.); kerstin.bauer@med.uni-tuebingen.de (K.B.); stephan.zipfel@med.uni-tuebingen.de (S.Z.); paul.enck@uni-tuebingen.de (P.E.); 2 Department of General, Visceral and Transplant Surgery, University Hospital, 72072 Tübingen, Germany; Rami.Archid@med.uni-tuebingen.de; 3 Institute for Medical Psychology and Behavioral Neurobiology, University Hospital, 72072 Tübingen, Germany; yvonne.ritze@uni-tuebingen.de; 4 Department of Medical Microbiology, School of Nutrition and Translational Research in Metabolism (NUTRIM) and Care and Public Health Research Institute(Caphri), Maastricht University Medical Centre, 6211 Maastricht, The Netherlands; j.penders@maastrichtuniversity.nl

**Keywords:** gastrointestinal microbiota, probiotics, bariatric surgery, psychological

## Abstract

The gastrointestinal (GI) microbiota plays an important role in health and disease, including brain function and behavior. Bariatric surgery (BS) has been reported to result in various changes in the GI microbiota, therefore demanding the investigation of the impact of GI microbiota on treatment success. The goal of this systematic review was to assess the effects of BS on the microbiota composition in humans and other vertebrates, whether probiotics influence postoperative health, and whether microbiota and psychological and behavioral factors interact. A search was conducted using PubMed and Web of Science to find relevant studies with respect to the GI microbiota and probiotics after BS, and later screened for psychological and behavioral parameters. Studies were classified into groups and subgroups to provide a clear overview of the outcomes. Microbiota changes were further assessed for whether they were specific to BS in humans through the comparison to sham operated controls in other vertebrate studies. Changes in alpha diversity appear not to be specific, whereas dissimilarity in overall microbial community structure, and increases in the abundance of the phylum Proteobacteria and *Akkermansia* spp. within the phylum Verrucomicrobia after surgery were observed in both human and other vertebrates studies and may be specific to BS in humans. Human probiotic studies differed regarding probiotic strains and dosages, however it appeared that probiotic interventions were not superior to a placebo for quality of life scores or weight loss after BS. The relationship between GI microbiota and psychological diseases in this context is unclear due to insufficient available data.

## 1. Introduction

Obesity and its associated comorbidities are a severe public health problem, with limited success achieved by conservative treatment approaches (lifestyle interventions) [1,2]. Bariatric surgery (BS) is currently the most effective method for patients with morbid obesity, with the most frequently performed procedures being the Roux-en-Y Gastric Bypass (RYGB) and Laparoscopic Sleeve Gastrectomy (LSG) [3]. Seeing as BS changes the anatomy and physiology of the gastrointestinal (GI) tract, it therefore may also affect the GI microbiota [2].

The GI microbiota is defined as the total amount of living microorganisms that colonize the GI tract of a host organism [4]. The GI microbiota live with the host in a complex and mutually beneficial relationship, playing a crucial role in the defense against external microorganisms, maintaining the physiology of the intestine, weight regulation and energy metabolism, nutrient/drug metabolism, and the production of metabolites, such as short-chain fatty acids (SCFA) which are involved in appetite regulation of the host [5]. SCFA may be involved in appetite regulation as they contribute to 5% to 10% of energy requirements of the host, and to influence satiety hormones such as Peptide YY via enteroendocrine cells and the gut–brain axis [1,6,7]. Dysbiosis of the GI microbiota was found to be associated with several pathologic conditions such as immune disorders, inflammatory bowel diseases, susceptibility to infections, type 2 diabetes mellitus, obesity, and hepatic and neurological states, among others [8].

The introduction of next-generation sequencing has instigated our insight into the microbiota with 16S rRNA gene amplicon sequencing being the predominant analysis technique for gut microbial composition (phylogenetic analysis) and allowing for a comprehensive description of these complex microbial communities [9,10]. In order to be able to compare the highly complex microbiota data of individuals, different ecological measures and indices are used. Alpha diversity describes the number of different species and their distribution in a microbial habitat and is thereby separated into richness and biodiversity [11]. Beta diversity describes the microbial community structure by the degree of similarity/dissimilarity of the microbial communities between different microbial habitats [12].

Although an “ideal” or “normal” gut microbiome is still unclear, many potential features have been identified, and conversely deviations from this have been associated with different pathophysiological conditions, such as obesity [1,13]. Seganfredo et al. reported that changes in the microbial composition occurred following weight loss after both restrictive diets and BS [1]. The microbiota is also affected by diet, with Western Diets (high in animal protein, fat and total energy, low in dietary fibers) being linked to reduced microbial diversity, beneficial bacteria and SCFA production. Current literature supports favorable changes in intestinal microbiota after BS with increases in alpha diversity, community structure and relative increases in the phyla Bacteroidetes and Proteobacteria, with a concomitant reduction in Firmicutes [14,15,16,17,18]. 

Similar to obesity, psychological conditions have been associated with inflammatory processes and dysbiosis in the GI microbiota [19,20]. Communication via the gut–brain axis is bidirectional and conducted through a complex network including the central nervous system (CNS), enteric nervous system and endocrine and immune systems [21]. The effects of the microbiota on brain development, psychological conditions and host behavior have attracted the attention of researchers over the last decade [19,22]. Therefore, changes in the GI microbiota may play an important role as diagnostic biomarkers for psychological conditions and individualized treatment pathways, or as targets to support or replace psychological therapies [23]. 

The GI microbiota may be influenced through the use of probiotics [24]. Preclinical studies addressing the benefit of probiotics for psychological states show a widely uniform picture, with improvements for stress, anxiety, and depression-associated behaviors reported [25,26,27,28,29], whereas the results in human trials are less clear, with mainly improvements concerning stress-related psychological problems currently observed in human volunteers [30,31,32]. Probiotic induced manipulation of the GI microbiota, through particular strains of *Lactobacillus* and *Bifidobacterium*, have been reported to have potential effects on CNS function via the gut–brain axis and may offer treatment options for the challenge of long-term obesity treatment and weight regulation; however, the efficacy of probiotic use in humans for obesity is controversial [33].

To date, no review has been conducted involving all of the following factors: GI microbiota changes in humans and other vertebrates following BS, the influence of probiotics on BS outcomes, and whether these influence patient psychology and behavior. This provokes the question, to what extent do GI microbiota change after BS influences psychological states and behavior? We therefore developed the following hypotheses:

- The GI microbial diversity will remain stable for richness and biodiversity (alpha diversity), but community structure will be dissimilar after BS compared to pre-surgery.
- Shifts in the abundance of specific microbial taxa will occur after BS, and the specificity of these changes in humans will be identified via comparison of different BS techniques to sham operations in vertebrate studies.
- The abundance of specific GI microbial taxa will shift after BS, and these changes will be associated with psychological and behavioral factors.
- The use of probiotics will influence outcomes after BS, including quality of life and psychological states.

## 2. Materials and Methods 

### 2.1. Literature Information Sources and Search Strategy

This review was developed and executed according to the Preferred Reporting Items for Systematic Reviews and Meta-Analyses (PRISMA) guidelines [34] to identify all relevant studies examining the GI microbiota and/or probiotic intervention in the context of BS in the databases PubMed and Web of Science as of 05.11.2018 and updated 18.11.2019. The protocol of this systematic review is registered at the PROSPERO platform with the registration number CRD42019119372. The full search strategy is documented in the Supporting Text S1 and consisted of three modules: type of BS, probiotics, and GI microbiota. Articles were screened for psychological or behavioral parameters during data organization. This was carried out as a secondary step to limit the risk of excluding relevant articles on BS and GI microbiota where psychological factors are not mentioned in the title, abstract, or keywords.

### 2.2. Eligibility Criteria

Eligibility criteria were based on the five PICOS dimensions, i.e., participants (P), interventions (I), comparators (C), outcome (O) and study design (S) [35]. 

Participants: adult patients with obesity and other vertebrates with obesity. No further restrictions regarding ethnicity, sex, or type of vertebrate were made. 

Intervention: BS and/or the use of probiotics after BS. Exclusion criteria included the additional use of antibiotics, prebiotics/symbiotics or other systemic interventions (e.g., proton pump inhibitors). 

Control: studies with or without control groups met eligibility criteria. 

Outcome measures: GI microbiota, and (secondary to this) assessment of psychological and behavioral factors. Studies were excluded if outcomes solely focused on pathogens (e.g., small intestinal bacterial overgrowth measured with H2-breath-test but not via GI microbiota).

Study design: prospective longitudinal, cross-sectional and retrospective studies, which were randomized and non-randomized, with any publication date and written in English and German. Only peer-reviewed, original articles were included. 

Eligibility for studies of Subgroup analysis: The eligibility criteria were kept broad to encapsulate the extent of the literature currently in this area. Subgroup analyses were then conducted to reduce heterogeneity of study designs and microbiota analysis techniques across studies. Eligibility criteria for the Subgroup 1 was studies from Group 1 (BS and microbiota in humans) with pre-post study design and 16S rRNA gene sequencing (*n* = 20); Subgroup 2 criteria was studies from Group 2 (BS and microbiota in other vertebrates) with sham operation/s as comparison and 16S rRNA gene sequencing (*n* = 21).

### 2.3. Study Selection, Data Collection and Organisation

The search results of the two databases were manually combined. Duplicates were removed and the titles and abstracts were screened to identify appropriate studies. Full-text articles were evaluated regarding their eligibility (CL and KB), with uncertainties being discussed between the authors (<3% cases). 

The studies were classified into three groups:

Group 1—BS and microbiota in humans
Group 2—BS and microbiota in other vertebrates
Group 3—BS and probiotics

Subgroups were developed to provide a more homogenous summary of findings.

Subgroup 1—Pre-post BS comparisons in human
Subgroup 2—BS to sham operation comparisons in other vertebrates

### 2.4. Data Items and Statistics

The following information was extracted from each included article for Groups 1, 2, and 3: study characteristics, BS type and probiotic intervention where appropriate, methods of gut microbiota analysis and outcomes, and all data available on psychological factors. The main outcomes concerning the GI microbiota were alpha diversity (richness and biodiversity), community structure/beta diversity, and microbial composition. For microbial abundance outcomes which were tested and not specifically referred to in text, no effect was presumed and written as not significant. Secondary outcomes including anthropometric, clinical, and behavioral changes were also retrieved. Authors were contacted if parameters for data extraction were missing. A total of 46% (13 out of 28) of authors provided the requested data.

Significant differences for alpha diversity, community structure, and taxonomy abundances were summarized in the two subgroups.

### 2.5. Risk of Bias

For all included studies a risk of bias assessment was performed using The Office of Health Assessment and Translation (OHAT) Risk of Bias rating tool for Human and Animal Studies [36]. This method was chosen because the same rating system can be applied to human and other vertebrate studies with different study designs. Depending on the study type, different (applicable) items are evaluated as described in the OHAT checklist. The items focus on the possible risk areas of adequate randomization, allocation to appropriate comparison groups, accounting for confounding and modifying variables, and confidence in the exposure and outcome assessment, among others. The rating ranged between: definitely low (“++”), probably low (“+”), probably high (“−” or “NR”: not reported), definitely high risk of bias (“−−”), or “NA”: not applicable. In addition, the 16S rRNA sequencing platforms and targeted hypervariable regions were assessed to address the bias of laboratory methods.

## 3. Results

**Figure 1:**
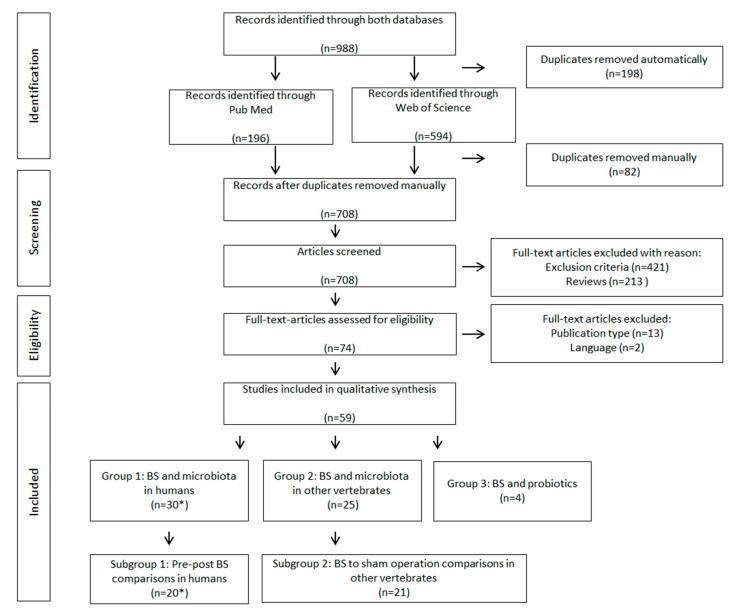
Flow chart of Preferred Reporting Items for Systematic Reviews and Meta-Analyses (PRISMA) systematic search. * 2 of the identified studies presented the same data and are discussed as a single study.

The literature search process used to identify eligible studies is shown in Figure 1. Out of 988 identified studies from the electronic databases, 59 studies remained for qualitative analysis. Thirty articles were categorized into group 1 (BS and microbiota in humans). The work by Campisciano et al. [37,38] is included as two original articles, but discussed and summarized as one due to presenting identical results. Twenty-five articles were categorized into group 2 (BS and microbiota in other vertebrates) and four articles were categorized into group 3 (BS and probiotics). All identified probiotic studies were conducted in humans.

### 3.1. Summary of Study Characteristics

Study characteristics across are summarized in Table 1 and Table 2, with additional information in single-study breakdown detailed in Supporting Table S1. The included studies consisted of randomized controlled trials (Group 1: *n* = 6; Group 2: *n* = 15; Group 3: *n* = 3), non-randomized controlled trials (Group 1: *n* = 11; Group 2: *n* = 9; Group), experimental designs (Group 1: *n* = 9; Group 2: *n* = 1), randomized (with no control) designs (Group 1: *n* = 2; Group 2: *n* = 1; Group 3: *n* = 1) and retrospective studies (Group 1: *n* = 1). Studies in humans included either both sexes (*n* = 26) or only females (*n* = 7). Studies conducted in other vertebrates were exclusively conducted in male animals (*n* = 22), except two. The surgical intervention type was highly diverse among the human and other vertebrate trials, however the most common were RYGB (*n* = 31) and LSG (*n* = 20). In 19 studies more than one surgical procedure was performed. 

Out of the 59 studies, 36 studies applied a pre-post design (baseline measurements provided) (Group 1: *n* = 23 [37,38,39,40,41,42,43,44,45,46,47,48,49,50,51,52,53,54,55,56,57,58,59]; Group 2: *n* = 10 [60,61,62,63,64,65,66,67,68,69]; Group 3: *n* = 4 [70,71,72,73]). Sixteen studies used a cross-sectional design (Group 1: *n* = 6 [74,75,76,77,78,79]; Group 2: *n* = 10 [80,81,82,83,84,85,86,87,88,89]), whereas four studies applied a longitudinal study design with multiple measurement points postoperatively but without baseline measurements (Group 2 [90,91,92,93]). Two studies did not report the study time points of sampling adequately [94,95].

### 3.2. Summary of Study Outcomes

Changes in GI microbiota was the primary outcome for 24 studies in Group 1 [37,38,39,40,41,42,44,45,47,48,49,51,52,53,54,55,56,57,59,75,76,77,78,79,94] and 12 studies in Group 2 [61,62,64,65,68,82,85,86,88,90,92,95]. For the remaining studies, primary outcomes surrounding clinical, anthropometric and metabolic parameters were reported (Group 1: *n* = 5 [43,46,50,58,74]; Group 2: *n* = 13 [60,63,66,67,69,80,81,83,84,87,89,91,93]). For Groups 1 and 2, outcome results including microbiota diversity and taxonomy changes are available in detail in Supporting Table S2. The outcomes for Group 3 included clinical, anthropometric and/or metabolic parameters [70,72,73] and Quality of Life (QoL) [71] and are detailed in Supporting Table S3. Analysis of microbiota samples were completed predominantly via next generation sequencing of 16S rRNA gene amplicons (Group 1: *n* = 23; Group 2: *n* = 22; Group 3: *n* = 1), with largely the hypervariable regions V3 and V4 targeted (detailed across studies below in the risk of bias section and for individual studies in Supporting Table S1).

### 3.3. Overview of Microbiota Changes Following BS in Humans (Group 1) and Other Vertebrates (Group 2)

Alpha diversity was reported to remain stable (richness: *n* = 6; biodiversity: *n* = 7) or increase (richness: *n* = 10; biodiversity: *n* = 7) in humans after BS, and to not significantly differ in BS compared to sham operated vertebrates in non-human studies (richness: *n* = 6; biodiversity: *n* = 5). Dissimilarity in microbial community structure was reported by studies in both groups (Group 1: *n* = 16; Group 2: *n* = 10). Changes in specific microbial taxa abundances were reported by studies in both groups; however, changes reported at the genus and species level were not consistent across studies and at phylum level abundances predominantly remained stable following BS.

Due to the heterogeneity in outcome comparisons and microbiota analysis techniques used, it was not feasible to summarize these groups’ findings clearly in figures. This led to the breakdown into Subgroup 1 and 2, as detailed in the Methods section. We noted the overall findings of the subgroup analyses aligned with the whole groups’ analyses, whilst being more practical for comparative summaries and figures. Therefore, further detail will now be provided for Subgroup 1 and 2, with full Group 1 and 2 Results discussed in the Supporting Text S2 and Supporting Table S2.

### 3.4. Subgroup Analysis

**Figure 2:**
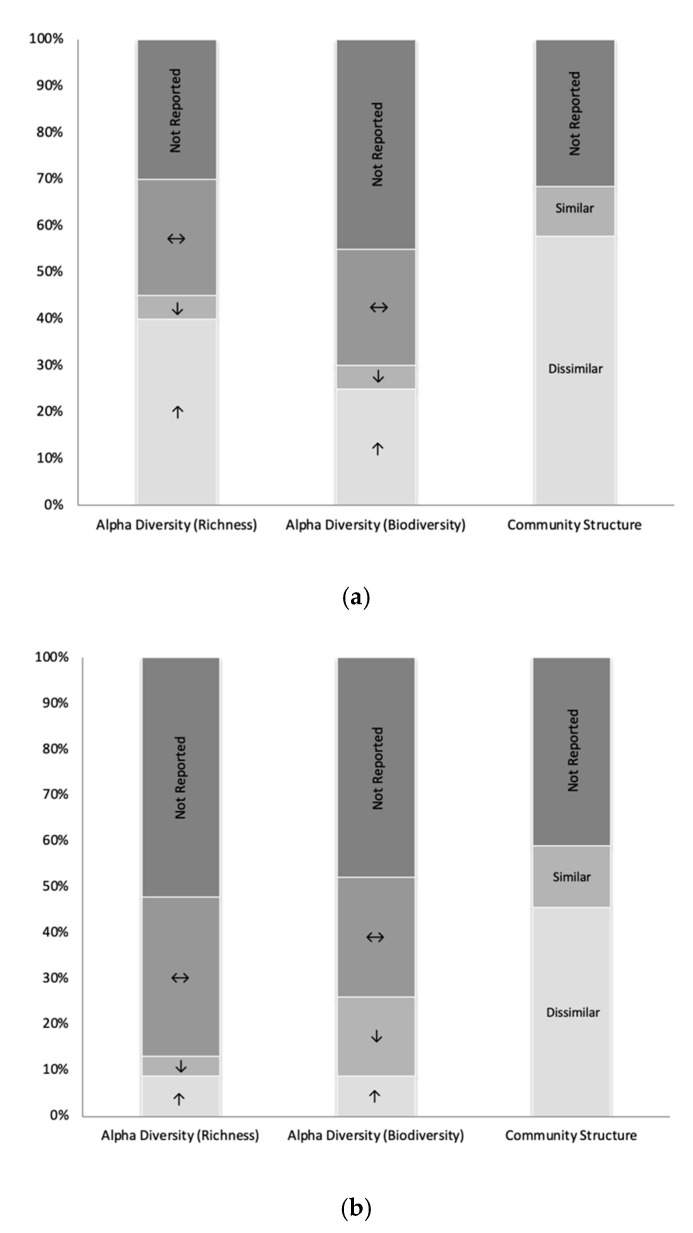

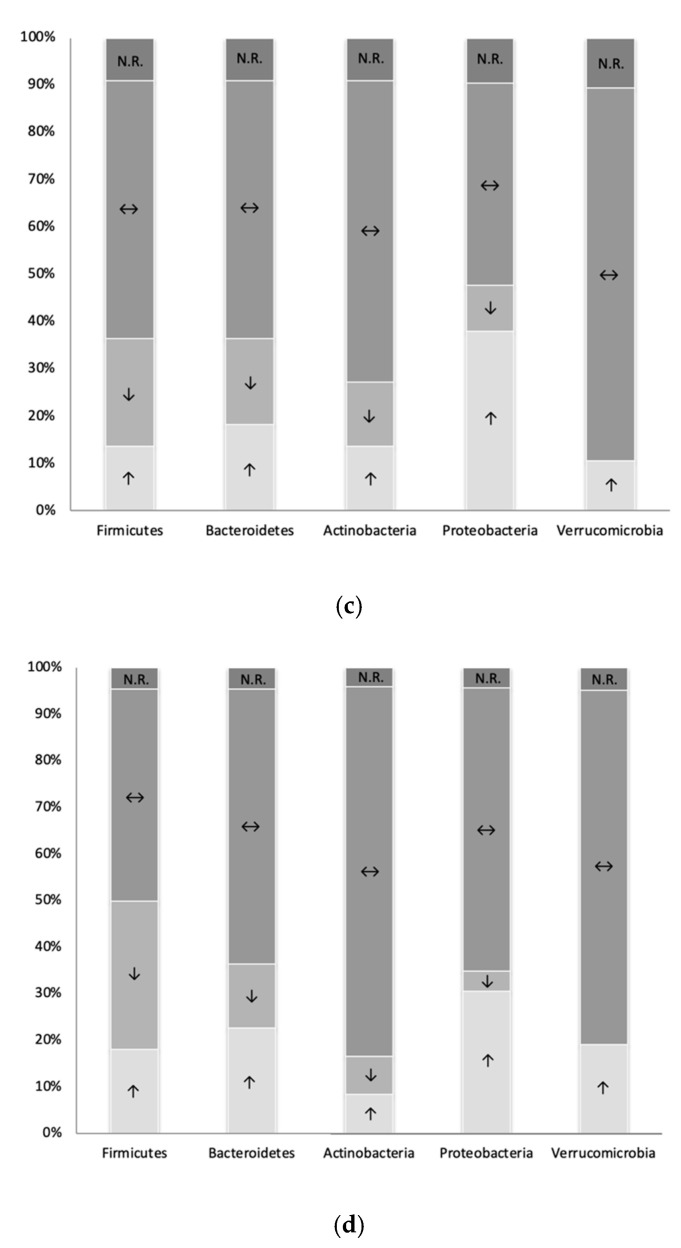
Microbiota changes in the context of bariatric surgery. (**a**) Subgroup 1 diversity changes; (**b**) Subgroup 2 diversity changes; (**c**) Subgroup 1 phylum changes; (**d**) Subgroup 2 phylum changes. ↑: Increase/higher; ↓: Decrease/lower; ↔: Stable/nil difference; N.R.: Not reported.

Frequency analyses for significant changes in alpha diversity, community structure and taxonomy were conducted in 2 subgroups; Pre-post BS comparisons in humans (Subgroup 1) and BS to sham operation comparisons in other vertebrates (Subgroup 2). This is summarized in Table 3 and shown in Figure 2. Additional information is available in Supporting Table S4.

#### 3.4.1. Subgroup 1 (Pre-Post BS Comparisons in Humans)

Data are summarized in Table 3 and Figure 2a,c. Eight studies reported an increase and five found no significant change for microbial richness in patients after surgery. The microbial biodiversity was also reported to predominantly increase (*n* = 5) or remain stable (*n* = 5). A decrease in alpha diversity was only reported by Patrone et al. [57]. The impact of BS on microbial community structure (beta-diversity) was examined in thirteen studies in Subgroup 1, with eleven studies observing significant dissimilarities between pre- and post-surgery microbial communities. It was found that phyla largely remained stable after BS when compared to baseline (Firmicutes *n* = 12; Bacteroidetes *n* = 12; Actinobacteria *n* = 14; Proteobacteria *n* = 9; Verrucomicrobia *n* = 15). However, Proteobacteria phylum was found to significantly increase in eight studies (RYGB *n* = 7; LSG *n* = 2; AGB *n* = 1; BIB *n* = 1). At the species level, *Akkermansia muciniphila* of the Verrucomicrobia phylum reported a significant increase following BS (*n* = 6). It was not apparent why in most studies the increase of *Akkermansia* spp. was not accompanied by an increase of Verrucomicrobia at phylum level, seeing as there are hardly any species other than *Akkermansia* spp. within this phylum in the human gut. Finally, *Escherichia coli* within Proteobacteria also increased in three studies after BS.

#### 3.4.2. Subgroup 2 (BS to Sham Operation Comparisons in Other Vertebrates)

Data are summarized in Table 3 and Figure 2b,d. Alpha diversity (richness and biodiversity was predominantly reported as comparable between BS and sham operation groups in animals (richness *n* = 6; biodiversity *n* = 5). The microbial community structure between groups was classified as dissimilar by ten of twelve reporting studies. Taxonomy abundances at the phylum level comparing BS to sham operated controls were largely found to not significantly differ between groups. Further, Firmicutes, Bacteroidetes, and Actinobacteria had mixed outcomes when a significant difference was reported. Proteobacteria and Verrucomicrobia were both higher following BS than following sham operations when a difference was reported (Proteobacteria *n* = 6; Verrucomicrobia *n* = 3). At class level, Gammaproteobacteria was higher after BS compared to sham operated controls in five studies. At genus level, *Bifidobacterium* within Actinobacteria (*n* = 3) and *Akkermansia* within Verrucomicrobia (*n* = 3) were reported to be higher after BS compared to sham.

#### 3.4.3. Similarities and Differences Identified between Subgroup 1 and Subgroup 2

Through the assessment of microbiota changes in other vertebrates comparing BS to sham operations the potential specificity of microbiota changes can be discussed. Alpha diversity (richness and biodiversity) was mostly found to not significantly differ after BS compared to sham operations in vertebrates and was frequently reported as stable in human studies; therefore, changes in alpha diversity may be not specific to BS in humans. Dissimilarity in community structure was consistently reported in human and other vertebrate studies, supporting specificity after BS. The increased abundances after BS reported in the human studies for the phylum Proteobacteria and *Akkermansia spp.* within Verrucomicrobia may be specific to BS as they were also identified in greater abundance in the BS operated groups than sham operated animals. With respect to commonly investigated phyla, Firmicutes, and Bacteroidetes, specificity of BS cannot be stated.

### 3.5. Influence of GI Microbiota and BS on Psychological and Behavioural Outcomes

Of the included studies, no investigations or outcomes on psychological states were reported. 

In Group 1 (BS and microbiota in humans), two studies reported outcomes at a behavioral level [56,58]. Sanmiguel et al. reported a decrease in hedonic hunger and decreased preference for energy-dense foods after LSG, which they were able to correlate to microbiota changes [58]. They found preference for energy-dense food to be negatively associated with *Akkermansia* abundance, and hedonic hunger to be negatively associated with *Butyricimonas*, *Enterococcus,* and *Odoribacter* and positively associated with *Anaerostipes* abundances. Similarly, Palmisano et al. reported a decreased preference for carbohydrate-dominant food items at after LSG; however, no correlations with microbiota were assessed [56].

In Group 2 (BS and microbiota in other vertebrates), Jahansouz et al. investigated whether social isolation in mice influences the GI microbiota and prevents successful weight loss compared to co-housed mice and found no difference post LSG [61]. However, interestingly, they found GI microbiota differences between sham operated co-housed and sham operated individually-housed mice. 

### 3.6. Influence of Probiotics on BS Outcomes (Group 3)

A total of four studies were included, of which three included a comparison group [70,71,72,73]. Probiotics were provided in capsule form [71,72,73], with three studies using single-strain probiotics (*Clostridium butyrium* [70], *Bifidobacterium longum BB536* [70], *Bacillus coagulans* [71], *Lactobacillus* [73]) and one study using the multi-strain probiotic *Bio-25 (Supherb*) [72]. The dosage ranged from containing 2.4 billion to >25 billion bacteria per capsule, and the duration of the treatments ranged from fourteen days to six months. Due to these differences in probiotic strains and dosages used between the studies, their findings cannot be directly compared as each probiotic strain has its own effect and possible outcome. However, outcomes were similar across studies. QoL was measured and found improvements, independent of type of surgery or control group treatments, across all studies, by all participant groups, and at all time points. Therefore, probiotic supplementation provided no additional benefit for QoL in these patients. 

Two studies assessed clinical parameters, reporting that differences in percentage body weight loss were not significant between probiotic and placebo groups after 6 months [72,73]. Sherf-Dagan et al. also investigated the GI microbiota of patients by 16S rRNA gene sequencing, which found an increased alpha diversity after LSG in both the probiotic and control groups [72]. Increases in the Firmicutes/Bacteroidetes ratio and the phyla Proteobacteria, Actinobacteria, and Verrucomicrobia were also shown in both probiotic and control groups. Therefore, this study concluded that probiotic use was not superior over the placebo following LSG in the context of GI microbiota changes.

### 3.7. Risk of Bias

The risk of bias for the studies included in the review conducted via the OHAT checklist is presented in Supporting Table S5. The randomization of trials was largely not applicable for studies in Group 1 (83%) and was of definitely or probably low risk for Group 2 (60%) and Group 3 (75%). There was definitely or probably a low risk of detection bias in 85% of studies (Group 1: 90%; Group 2: 76%; Group 3: 100%) and of reporting bias in 92% of studies (Group 1: 97%; Group 2: 92%; Group 3: 50%). Attrition bias was of definitely or probably low risk in human studies (Group 1: 50%; Group 3; 75%); however, it was of definitely or probably high risk for 36% of animal studies (Group 2). Furthermore, the adherence to study protocols was probably low risk of bias. 

Additional bias considerations specific to the microbiota analysis techniques include the differing methods for sampling, gene extraction, and sequencing used across the studies. The sequencing platforms (next generation sequencing of 16S rRNA gene amplicons: *n* = 46; including Illumina MiSeq *n* = 24; Thermofisher Ion Torrent *n*= 6; Illumina HiSeq *n* = 5; Roche GS-FLX *n* = 4; Sanger *n* = 2; SOLiD *n* = 1; Qiagen Kit *n* = 1), and the 16S rRNA hypervariable regions targeted for sequencing (V1: *n* = 5; V2: *n* = 7; V3: *n* = 14; V4: *n* = 27; V5: *n* = 3; V6: *n* = 5; V7-V9: *n* = 1) differed between studies. The choice of specific hypervariable region/s targeted in 16S rRNA sequencing has been shown to alter the microbiota taxa observed [96,97], and therefore multiple regions were targeted in some studies in attempts to reduce amplification bias (Group 1: *n* = 10; Group 2: *n* = 7). The information regarding microbiota samples/analysis for individual studies is presented in Supporting Table S1.

## 4. Discussion

This review included 59 articles and examined to what extent the GI microbiota changes after BS and whether these changes influence psychological states and behaviors. Overall, changes in GI microbiota were observed and reported following BS; however, the type and extent of the reported changes varied between studies. No data were available for microbiota changes related to psychological or behavioral factors after BS and probiotics had no additional benefit after BS. 

This review supports the first hypotheses that GI alpha diversity (richness and biodiversity) will remain stable and that community structure will be dissimilar for patients who have undergone BS. Microbial richness and biodiversity was not significantly different between BS and sham operation groups in other vertebrates (subgroup 2), whereas human study outcomes reported a mix of an increase or unchanged for both richness and biodiversity. This inconsistency in outcomes in human studies has also been noted in a review by Seganfredo et al. looking at microbiota changes after weight loss, where some studies found alpha diversity to increase and others to remain stable [1]. The results for community structure provided a uniform picture in humans and other vertebrates, with dissimilarities being seen both for comparisons between pre- and post-surgery in humans and between BS and sham operated controls in animals.

The type of surgical intervention influences the type and intensity of GI microbiota changes after BS and appears to be specific due to consistent findings for human and other vertebrate studies. The results of the included studies indicate that bypass procedures have a stronger effect on the GI microbiota than other techniques in both humans and other vertebrates. Due to the combination of nutrient restriction and malabsorption, bypass procedures impact more strongly on digestion and metabolism, and therefore may affect GI microbiota via multiple mechanisms, including accelerated transit time, increased exposure to gastric acid remnant, and a possible shift in small intestine residing microbiota to the large intestine [1,79,98]. Seeing as LSG and Adjustable Gastric Band (AGB) are restrictive procedures, they may cause fewer GI microbiota changes [18,42]. This was also highlighted in a recent review by Catoi et al. stating that microbiota changes following LSG may be the result of the associated caloric restriction which occurs pre- and post-BS [2]. The included study by Paganelli et al. additionally assessed the microbiota of patients after 2 weeks of recommended pre-surgery caloric restriction and found significant changes to the GI microbiota, some of which returned to baseline following surgery and the normalization of diet, supporting that some microbiota changes may be a result of caloric restriction [53]. However, all studies showed that BS had an effect on the GI microbiota, with RYGB inducing greater functional and taxonomic changes.

Changes in the abundance of a few specific microbiota taxa were reported, supporting the second hypothesis; however, changes reported at the genus and species level were not consistent across studies and at the phylum level abundances predominantly remained stable following BS. A notable change in microbiota abundance observed by the included studies was the increase in the species *Akkermansia muciniphila* after BS. Higher abundancies of *Akkermansia* spp. have been observed in lean or normal weight versus humans/vertebrates with obesity [99,100]. In addition, weight loss is accompanied by an increase in *Akkermansia* spp. [1,99,101] and high abundances have also been reported in patients with anorexia nervosa [7,102], therefore, suggesting that *Akkermansia* spp. favour a habitat with decreased food intake. This is also supported by the findings that weight gain is accompanied by a decrease in *Akkermansia* spp. [102,103].

Although studies in humans and other vertebrates were included in this review, it is important to acknowledge that their results are not directly comparable. This is due to the differing physiology of species and differing comparison groups; however, through the inclusion of Subgroup 2 (BS to sham operation comparisons in other vertebrates) the potential specificity of microbiota changes can be discussed. Changes in alpha diversity (richness and biodiversity) appear not to be specific to BS, whereas dissimilarity in community structure was consistently reported in human and other vertebrate studies, supporting specificity after BS. The increase reported in human studies for the phylum Proteobacteria and *Akkermansia* spp. within Verrucomicrobia may be specific to BS, rather than a consequence of the trauma of surgery. Changes in microbiota taxa were observed at a greater extent in other vertebrates following BS than sham operations. However, it is important to note that changes in the GI microbiota were also observed in sham operated animals, supporting the thought that surgery itself may have short-term impacts on the GI microbiota. For ethical reasons, only a few studies worldwide have performed sham surgery in humans [104,105,106]. However, the reported microbial changes detailed in the studies of Subgroup 2 (BS to sham operation comparisons in other vertebrates) demonstrate some specificity of changes following BS. 

Our third hypothesis looked at potential correlations between microbial patterns and psychological states and behavior, supported by the growing literature on the gut–brain axis’ contribution, especially with respect to mental illnesses and disturbed GI microbiota [8,20]. In the included studies, there were only outcomes relating to food preferences/hedonic desires [55,58]. Sanmiguel et al. observed a significant relationship between decreased preference for energy-dense food and an increased abundance of *Akkermansia* spp. [58]. This aligns with the suggestion that *Akkermansia* spp. favor a habitat with decreased food intake; however, the direction of this relationship remains unclear. No outcomes relating GI microbiota to anxiety, depression, or other mental conditions were reported. BS has previously been linked with post-operative reduction in depressive and stress symptoms in long-term follow-up studies [107,108]. However, an association of psychological states with the GI microbiota remains unclear from the included studies. This also allows the conclusion that changes in psychological state after BS may play an indirect role (such as via altered eating behavior) rather than directly on the GI microbiota. Nevertheless, the potential remains for GI microbiota changes to influence psychological states and behaviors in the context of BS, mediated by the gut–brain axis and reflected in the higher prevalence of anxiety and depression in patients with obesity.

Against our final hypothesis, the use of probiotic supplementation after BS was not superior over placebo in the included studies. The impact of probiotics following BS was hypothesized to influence the outcomes of BS and psychological factors due to the fact that some probiotics have demonstrated effects on the CNS [20,109,110]. Out of four studies, three used strains of *Lactobacillus* spp. and two used strains of *Bifidobacterium* spp. for their investigations. Specific strains of *Lactobacillus* spp. and *Bifidobacterium* spp. may be capable of effecting mood, anxiety and cognition via the gut–brain axis [19,109,111,112]; however, this remains unclear. Further research is needed to assess the impact of specific probiotic strains on mood, stress resilience, and psychological states such as anxiety and depression, and for in the context of BS in potential psychologically unstable patients [110].

## 5. Strengths and Limitations

This review involved a broad search for aspects of the GI microbiota in the context of BS, including probiotics, to ensure that all possible literature for the connection between psychological factors and microbiota after BS would be found. The specific approach taken with the subgroup analyses is a special strength of this review. The novel approach to summarize usually very heterogenetic data, made it possible to better summarize the current literature on changes in GI microbiota after BS through pre-post comparisons in humans (Subgroup 1). This additionally allowed for the comparison with vertebrate studies involving sham operated controls (Subgroup 2), to discuss which changes in the GI microbiota may be specific or non-specific to BS. A high response rate from study authors was achieved (46%), minimizing missing information. 

However, there are also limitations to this work. Specifically, the inability to control for potential confounding variables which may result in microbiota changes such as dietary choices, eating behaviors, and other environmental factors in human studies. Another important consideration for microbiota changes in the context of BS is the various protocols involved with the surgery, specifically the surgical guidelines’ recommendation for prophylactic antibiotics preoperatively in GI surgeries, including BS, which would be expected to influence the GI microbiota [113,114]. Additionally, the specific strains of probiotics tested in the included studies were inconsistent and have not been shown to act on the CNS in humans.

## 6. Conclusions

In this review, the GI microbiota in the context of BS were investigated. BS was found to be associated with changes in the diversity and taxonomy of the GI microbiota. The observed dissimilarity in community structure, increases in the phylum Proteobacteria and *Akkermansia* spp. within Verrucomicrobia after BS may be specific to BS in humans. Human probiotic studies found probiotic interventions not to be superior to placebo for QoL scores or body weight loss after BS. It was also hoped to investigate whether outcomes relating to psychology and behaviors were correlated to GI microbiota changes, due to the influence of the gut–brain axis and the typically higher prevalence of anxiety and depression in patients with obesity. Further research is required in this area, and also to decipher whether the link between changes in GI microbiota and changes in psychological states after BS are related directly, or indirectly, via the changes in eating behavior and diet observed after BS. 

## Figures and Tables

**Table 1:** Characteristics across human studies—Groups 1 and 3.

Follow up after Surgery ^1^ (Months)	Median	IQR	Minimum	Maximum
All (*n* = 30)	6.0	[0.5–24.0]	0.5	112.8
BS and microbiota in humans (*n* = 26)	6.0	[5.3–12.0]	1.0	112.8
BS and probiotics in humans (*n* = 4)	4.5	[2.4–6.0]	0.5	6.0
**Sample size**				
All (*n* = 33)	24.0	[14.0–45.0]	6.0	267.0
BS and microbiota in humans (*n* = 29)	21.0	[13.0–43.0]	6.0	267.0
BS and probiotics in humans (*n* = 4)	52.0	[43.0–70.0]	40.0	100.0
**Age of Study Population ^2^ (Years)**				
All (*n* = 27)	43.5	[39.0–47.8]	23.5	51.5
BS and microbiota in humans (*n* = 23)	43.5	[39.0–47.8]	23.5	51.5
BS and probiotics in humans (*n* = 4)	43.4	[40.2–45.7]	35.1	48.0
**Sexes of Study Population ^3^**	**Both Sexes**	**Only Females**	**Only Males**	
All (*n* = 33)	*n* = 26	*n* = 7	*n* = 0	
BS and microbiota in humans (*n* = 29)	*n* = 22	*n* = 7	*n* = 0	
BS and probiotics in humans (*n* = 4)	*n* = 4	*n* = 0	*n* = 0	
**BMI: Categorization ^4,5^**	**Overweight (25–< 29.9)**	**Obesity Class 1 (30–< 34.9)**	**Obesity Class 2 (35–< 39.9)**	**Obesity Class 3 (≥ 40)**
All (*n* = 30)	*n* = 6	*n* = 9	*n* = 13	*n* = 23
BS and microbiota in humans (*n* = 26)	*n* = 6	*n* = 9	*n* = 13	*n* = 19
BS and probiotics in humans (*n* = 4)	*n* = 0	*n* = 0	*n* = 0	*n* = 4
**Diabetes Status ^6^**	**Only Patients with Diabetes**	**Patients with and without Diabetes**	**No Patients with Diabetes**	
All (*n* = 24)	*n* = 4	*n* = 15	*n* = 5	
BS and microbiota in humans (*n* = 21)	*n* = 4	*n* = 12	*n* = 5	
BS and probiotics in humans (*n* = 3)	*n* = 0	*n* = 3	*n* = 0	
**Type of Surgery ^7^**	**RYGB**	**LSG**	**AGB**	**Other ^8^**
All (*n* = 33)	*n* = 20	*n* = 12	*n* = 3	*n* = 8
BS and microbiota in humans (*n* = 29)	*n* = 18	*n* = 10	*n* = 3	*n* = 7
BS and probiotics in humans (*n* = 4)	*n* = 2	*n* = 2	*n* = 0	*n* = 1

^1^ Two studies did not report their exact study length; ^2^ Four studies did not report the exact age of study population; ^3^ Three studies did not appropriately specified the sexes; ^4^ Some studies included patients of different BMI categorizations; ^5^ Two studies did only report the weight; ^6^ Seven studies did not appropriately specified the diabetes status; ^7^ Some studies included different types of surgery; ^8^ BIB, DJB, LGB, Mini Gastric bypass, Jejunoileostomy.

**Table 2:** Characteristics across other vertebrates’ studies—Group 2.

Study Length ^1^ (Weeks)	Median	IQR	Minimum	Maximum
BS and microbiota in other vertebrates (*n* = 23)	9.0	[5.3–12.0]	2.0	24.0
**Sample size ^2^**				
BS and microbiota in other vertebrates (*n* = 21)	21.0	[17.5–30.5]	6.0	100.0
**Ages of Animals ^3^ (Weeks)**				
BS and microbiota in other vertebrates (*n* = 17)	8.0	[6.0–10.0]	4.0	80.0
**Sexes of Animals ^4^**	**Both Sexes**	**Only Females**	**Only Males**	
BS and microbiota in other vertebrates (*n* = 24)	*n* = 1	*n* = 1	*n* = 22	
**Species of Animal**	**Rats**	**Mice**	**Dogs**	
BS and microbiota in other vertebrates (*n* = 25)	*n* = 19	*n* = 5	*n* = 1	
**Diabetes Status**	**Only Animals with Diabetes**	**Animals with and without Diabetes**	**No Specific Information/Test**	
BS and microbiota in other vertebrates (*n* = 25)	*n* = 6	*n* = 4	*n* = 15	
**Type of Surgery ^5^**	**RYGB**	**LSG**	**DJB**	**Other ^6^**
BS and microbiota in other vertebrates (*n* = 25)	*n* = 11	*n* = 8	*n* = 6	*n* = 7

^1^ One study did not report its exact study length; ^2^ Three studies did not report their exact sample size; ^3^ Eight studies did not report the exact ages; ^4^ One study did not report the sexes of the animals; ^5^ Some studies included more than one surgery type; ^6^ IT, GG, BL, DES, BPD/DS.

**Table 3:** Subgroup analysis outcomes for alpha diversity, community structure, and taxonomy changes at the phylum level.

Subgroup 1: Pre-Post BS Comparisons in Humans																		
Author (Year)	Surgery Type	Alpha D. Richness			Alpha D. Biodiversity			Community Structure		Firmic.		Bactero.		Actinob.		Proteob.		Verruco.
*Campisciano (2017/18)*	Bypass/LSG	↔			N.R.			N.R.		↑	↓	↓	↔	↓		↑	↓	↔
*Chen (2017)*	RYGB	N.R.			N.R.			N.R.		↔		↑		↔		↔		↔
*Cortez (2018)*	DJB	↑			↑			Dis		↓		↑		↔		↔		↑
*Damms-Machado (2014)*	LSG	N.R.			N.R.			Dis		↓		↑		↔		↔		↔
*Graessler (2013)*	RYGB	N.R.			N.R.			N.R.		↓		↓		↓		↑		↑
*Kellerer (2019)*	LSG	↑			↑			Sim		↔		↔		↔		↔		↔
*Kong (2013)*	RYGB	↑			N.R.			N.R.		N.R.		N.R.		N.R.		N.R.		N.R.
*Lee (2019)*	RYGB/AGB	N.R.			N.R.			Sim		↔		↔		↑	↔	↑		↔
*Lin (2019)*	LSG	↑			N.R.			N.R.		↔		↔		↔		↔		↔
*Liu R.X (2017)*	LSG	↑			N.R.			Dis		N.R.		N.R.		N.R.		N.R.		N.R.
*Medina (2017)*	RYGB/LSG	N.R.			N.R.			Dis		↔	↑	↔	↓	↑	↔	↑		↔
*Murphy (2017)*	RYGB/LSG	↑		↔	↑		↔	N.R.		↑	↔	↓	↑	↑	↔	↔		↔
*Paganelli (2019)*	RYGB/LSG	N.R.			↔			Dis		↔		↔		↓		↑		↔
*Pajecki (2019)*	RYGB	↔			↔			Dis *		↔		↔		↔		↓		↔
*Palleja (2016)*	RYGB	↑			↑			Dis		↔		↔		↔		↑		↔
*Palmisano (2019)*	RYGB/LSG	↔			↔			Dis		↔		↔		↔		↑	↔	↔
*Patrone (2016)*	BIB	↓			↓			Dis		↔		↔		↔		↑		↔
*Sanmiguel (2017)*	LSG	↔			↔			Dis		↓		↔		↔		↔		↔
*Wang (2019)*	RYGB/LSG	↑			↑			Dis		↔		↔		↔		↔		↔
**Subgroup 2**: BS to sham operation comparisons in other vertebrates																		
**Author (Year)**	**Surgery Type**	**Alpha D.** **Richness**			**Alpha D.** **Biodiversity**			**Community Structure**		**Firmic.**		**Bactero.**		**Actinob.**		**Proteob.**		**Verruco.**
*Alvarez (2018)*	LSG1/LSG2	↔			↔			N.R.		↔		↔		↔	↑	↔		↔
*Basso (2016)*	GG	↔			↑			Dis		↔		↔		↔		↔		↔
*Cummings (2013)*	IT	N.R.			N.R.			N.R.		↔		↔		↔		↔		↔
*Duboc (2017)*	RYGB/LSG	↔			↔			Dis		↔		↔		↔		↔		↔
*Guo (2016)*	RYGB/LSG	↔			↑			Dis		↑	↓	↔	↑	↔	↑	↑	↔	↔
*Huang (2014)*	LSG	N.R.			N.R.			N.R.		↔		↔		↔		↔		↔
*Huh (2019)*	RYGB/LSG	↑			N.R.			Dis		↓		↔		↔		↑		↔
*Jahansouz (2017)*	LSG (*A*/*B*)	↔			↔			Sim		↓		↑		↔	↓	↔		↔
*Jiang (2016)*	DJB	N.R.			↓			Dis		↑		↓		↓		↓		↑
*Kashihara (2015)*	DJB	N.R.			N.R.			N.R.		↔		↔		↔		↔		↔
*Kim (2017)*	DES	N.R.			N.R.			Sim		↔		↓		↔		↔		↑
*Li J.V. (2011)*	RYGB	N.R.			N.R.			N.R.		↑		↔		↔		↑		↔
*Li S. (2017)*	DJB/LSG	N.R.			N.R.			N.R.		N.R.		N.R.		N.R.		N.R.		N.R.
*Liou (2013)*	RYGB	N.R.			N.R.			Dis		↓		↑		↔		↑		↑
*Liu (2018)*	RYGB	N.R.			↔			Dis		↓		↑		↔		↔		↔
*Miyachi (2017)*	B-DJB/J-DJB	N.R.			N.R.			N.R.		↔		↔		↔		↔		↔
*Mukorako (2019)*	BPD/DS/DS/LSG	↔	↓	↔	↓	↓	↔	Dis		↔		↔		↔		↔		↔
*Shao (2017)*	RYGB/LSG	N.R.			↓			Dis	Sim	↔		↔		↔		↑	↔	↔
*Shao (2018)*	LSG	↑			↔			Dis		↓		↑		↔		↔		↑
*Wang (2019)*	RYGB	N.R.			N.R.			N.R.		↓		↔		↔		↑		↔
*Zhang (2015)*	DJB	↔			N.R.			N.R.		↑		↓		↔		↑		↔

* Dissimilarity in unweighted UniFrac (*p* = 0.029), trend to dissimilarity in weighted UniFrac (*p* = 0.08). *A*: Individually housed mice, *B*: Cohoused mice. ↑: Increase/higher; ↓: Decrease/lower; ↔: Stable/nil difference; N.R.: Not reported.

## Supplementary Materials

The following are available online at https://www.mdpi.com/2072-6643/12/8/2396/s1, Text S1: Search strategy, Table S1: Overview of individual study characteristics, Text S2: Result summaries of Group 1 and Group 2 outcomes, Table S2: Microbiota changes following bariatric surgery (Group 1, Group 2), Table S3: Group 3—Bariatric surgery and probiotics, Table S4: Subgroup microbiota changes results, Table S5: OHAT Risk of Bias.

## Abbreviations

AGB	Adjustable gastric banding
BIB	Bilio–intestinal bypass
BL	Blind loop
BMI	Body mass index
BPD/DS	Bilopancreal diversion with duodenal switch
BS	Bariatric surgery
CNS	Central nervous system
DES	Duodenal endoluminal barrier sleeve
DJB	Duodenal–jejunal bypass (B-DJB: with biliopancreatic limb, J-DJB: with jejunectomy)
DS	Duodenal switch
GG	Glandular gastrectomy
GI	Gastrointestinal
IQR	Interquartile range
IT	Ileal interposition
LSG	Laparoscopic sleeve gastrectomy
LGB	Laparoscopic gastric bypass
N.R.	Not reported
QoL	Quality of life
RYGB	Roux-en-Y gastric bypass
SCFA	Short chain fatty acids
VBG	Vertical banded gastroplasty
