# Supplementary material for: Gut Microbiota, Probiotics and Psychological States and Behaviors after Bariatric Surgery—A Systematic Review of Their Interrelation
Source: Nutrients. 2020 Aug 10;12(8):2396. doi: 10.3390/nu12082396 (PMC7468806; doi:10.3390/nu12082396)
Supplement: Supplementary file 1 [file nutrients-12-02396-s001.pdf]

Gut microbiota, probiotics and psychological states and behaviours after bariatric surgery – a systematic review of their interrelation.

Jessica Cook[1*], Christine Lehne[1*], Alisa Weiland[1], Rami Archid[2], Yvonne Ritze[3], Kerstin Bauer[1], Stephan Zipfel[1], John Penders[4], Paul Enck[1], Isabelle Mack[1]

SUPPORTING INFORMATION:

Text S1: Search Strategy

PubMed-

((((((((((((((((((sleeve gastrectomy[Text Word]) OR gastric bypass surgery[Text Word]) OR RYGB[Text Word]) OR DJB[Text Word]) OR duodenal-jejunal bypass[Text Word]) OR jejunostomy[Text Word]) OR gastrectomy[Text Word]) OR jejunoileal bypass[Text Word]) OR gastric bypass[Text Word]) OR bariatric surgery[Text Word]) OR obesity/surgery[Text Word]) OR obesity surgery[Text Word]) OR gastrojejunostomy[Text Word])) AND ((probiotics[Text Word]) OR probiotic[Text Word]))) OR ((((((((((((((((((sleeve gastrectomy[Text Word]) OR gastric bypass surgery[Text Word]) OR RYGB[Text Word]) OR DJB[Text Word]) OR duodenal-jejunal bypass[Text Word]) OR jejunostomy[Text Word]) OR gastrectomy[Text Word]) OR jejunoileal bypass[Text Word]) OR gastric bypass[Text Word]) OR bariatric surgery[Text Word]) OR obesity/surgery[Text Word]) OR obesity surgery[Text Word]) OR gastrojejunostomy[Text Word])) AND microbiota; (((microbiota[Text Word]) OR microbiome[Text Word]) OR microflora[Text Word])) AND (((((((feces[Text Word]) OR gastrointestinal tract[Text Word]) OR gut[Text Word]) OR intestinal[Text Word]) OR gastrointestinal[Text Word]) OR fecal[Text Word]) OR faecal[Text Word]))))

Web of Science-

((TS=(sleeve gastrectom*) OR TS=(gastric bypass surger*) OR TS=(RYGB) OR TS=(DJB) OR TS=(duodenal?jejunal bypass*) OR TS=(jejunostom*) OR TS=(gastrectom*) OR TS=(jejunoileal bypass*) OR TS=(gastric bypass*) OR TS=(bariatric surger*) OR TS=(obesity surger*) OR TS=(gastrojejunostom*)) AND TS=(probiotic*)) OR ((TS=(sleeve gastrectom*) OR TS=(gastric bypass surger*) OR TS=(RYGB) OR TS=(DJB) OR TS=(duodenal?jejunal bypass*) OR TS=(jejunostom*) OR TS=(gastrectom*) OR TS=(jejunoileal bypass*) OR TS=(gastric bypass*) OR TS=(bariatric surger*) OR TS=(obesity surger*) OR TS=(gastrojejunostom*)) AND (TS=(microbiota*) OR TS=(microbiom*)) AND (TS=(feces) OR TS=(gastrointestinal tract) OR TS=(gut*) OR TS=(intestin*) OR TS=(gastrointestin*) OR TS=(fecal*) OR TS=(faecal*)))((TS=(sleeve gastrectom*) OR TS=(gastric bypass surger*) OR TS=(RYGB) OR TS=(DJB) OR TS=(duodenal?jejunal bypass*) OR TS=(jejunostom*) OR TS=(gastrectom*) OR TS=(jejunoileal bypass*) OR TS=(gastric bypass*) OR TS=(bariatric surger*) OR TS=(obesity surger*) OR TS=(gastrojejunostom*)) AND TS=(probiotic*)) OR ((TS=(sleeve gastrectom*) OR TS=(gastric bypass surger*) OR TS=(RYGB) OR TS=(DJB) OR TS=(duodenal?jejunal bypass*) OR TS=(jejunostom*) OR TS=(gastrectom*) OR TS=(jejunoileal bypass*) OR TS=(gastric bypass*) OR TS=(bariatric surger*) OR TS=(obesity surger*) OR TS=(gastrojejunostom*)) AND (TS=(microbiota*) OR TS=(microbiom*)) AND (TS=(feces) OR TS=(gastrointestinal tract) OR TS=(gut*) OR TS=(intestin*) OR TS=(gastrointestin*) OR TS=(fecal*) OR TS=(faecal*)))

Table S1: Overview of individual study characteristics

**Group 1:** Bariatric surgery and microbiota in humans				
**Author** (Year)	**Study Type/ Length**	**Sample Size and Characteristics**<br>n; Age (year) (SD); Sex %f, BMI [kg/m²] (SD)	**Surgery Type**	**Microbiota Samples / Analysis**
Aron-Wisnewsky (2018)	NRCT<br>12M	n=110; age: 39.9 (9.9); 100% f; BMI: 45.6 (5.2)<br>**i:** n=20 **ii:** n=41 **c:** n=49	**i:** AGB<br>**ii:** RYGB<br>**c:** CWL	Faecal samples<br>Shotgun metagenomics sequencing
Bjoerneklett (1981)	RCT<br>19M	**i:** n=9; age: 34; 100% f; BMI: N.R.<br>**ii:** n=9; age: 34; 89% f; BMI: N.R.<br>**iii:** n=8; age: 33; 88% f; BMI: N.R.<br>**c:** n=12	**i:** End-to-end jejunoileostomy<br>**ii:** End-to-side jejunoileostomy<br>**iii:** Gastric bypass<br>**c:** HWC	Jejunal fluid sample<br>Gas-solid chromatography
Campisciano (2018/2017)	NRCT<br>3M	n=40; age: N.R.; 78% f; BMI: 34.1 (4.1)<br>**i:** n=10 **ii:** n=10 **c:** n=20	**i:** LSG<br>**ii:** LGB<br>**c:** HWC	Faecal samples<br>16S rRNA gene sequencing (V1-V3) by Thermofisher Ion Torrent
Chen (2017)	exp.<br>6M	**i:** n=24; age: 40 (9); 0% f; BMI: N.R.	**i:** RYGB	Faecal samples<br>16S rDNA by PCR
Cortez (2018)	RCT<br>12M	**i:** n=11; age: 47 (8), N.R. f; BMI: 29.7 (1.9)<br>**c:** n=10; age: 44 (5), N.R. f; BMI: 31.7 (3.5)	**i:** DJB<br>**c:** CWL	Faecal samples<br>16S rRNA gene sequencing (V4) by Illumina MiSeq
Damms-Machado (2014)	NRCT<br>6M	**i:** n=5; age: 48 (3); 100% f; BMI: 45.8 (0.9)<br>**c:** n=5; age: 48 (3), 100% f; BMI: 40.2 (1)	**i:** LSG<br>**c:** Very low calorie diet	Faecal samples<br>SOLiD 16S rRNA gene sequencing and SOLiD shotgun sequencing
Federico (2016)	NRCT<br>6M	**i:** n=28; age: 47.8; 71.4% f; BMI: 51.5 (12.5)<br>**c:** n=28; age: 45; 60.7% f; BMI: <25	**i:** BIB<br>**c:** HWC	Faecal samples<br>PCR-DGGE
Fouladi (2019)	NRCT<br>N.R.	**i:** n=12; age: 42.1; 100% f; BMI: 32.7 (1.5)<br>**c:** n=6, age: 44.7, 100% f, BMI: 31.2 (2.4)	**i:** RYGB<br>**c:** ObC	Faecal samples<br>16S rRNA gene sequencing (V4) by Illumina MiSeq
Furet (2010)	NRCT<br>6M	**i:** n=30; age: 42 (2); 90% f; BMI: 48.3 (1.6)<br>**c:** n=13; age: 36 (3); N.R. f; BMI: 21.7 (0.4)	**i:** RYGB<br>**c:** HWC	Faecal samples<br>Real-time PCR
Graessler (2013)	exp.<br>3M	**i:** n=6; age: 46.0 (5); 50% f; BMI: 46.1 (4.0)<br>**c:** n=5; N.R.	**i:** RYGB<br>**c:** HWC	Faecal samples (paper-based stool collectors)<br>16S rRNA gene sequencing by Illumina MiSeq
Gutiutiérrez-Repiso (2019)	exp.<br>6M	**i:** n=24; age: 46.4 (2.4); 85.7% f; BMI: N.R.	**i:** RYGB	Faecal samples<br>16S rRNA gene sequencing (V2-V4, V6-V9) by Thermofisher Ion S5
Ilhan (2017)	NRCT<br>N.R.	**i:** n=24; age: 51 (7); 67% f; BMI: 30.8<br>**ii:** n=14; age: 46 (11); N.R. f; BMI: 36.6<br>**c:** n=10; age: 42 (15);70% f; BMI: 22.3<br>**cc:** n=15; age: 50 (9); 53% f; BMI: 43.5	**i:** RYGB<br>**ii:** AGB<br>**c:** HWC<br>**cc:** ObC	Faecal samples<br>16S rRNA gene sequencing (V4-V6) by Illumina MiSeq
Kellerer (2019)	NRCT<br>6M	**i:** n=8; age: N.R.; N.R. f; BMI: 45.8<br>**c:** n=8; N.R.	**i:** LSG<br>**c:** HWC	Faecal samples<br>16S rRNA gene sequencing (V3-V4) by Illumina MiSeq
Kong (2013)	exp.<br>6M	**i:** n=30; age: N.R.; 100% f; BMI: N.R.	**i:** RYGB	Faecal samples<br>16S rRNA gene sequencing (V3-V4) by Roche GS-FLX 454
Lee (2019)	RCT<br>9M	**i:** n=4; age: 57; 100% f; BMI: 35.1<br>**ii:** n=4; age: 45; 100% f; BMI: 35.8<br>**c:** n=4; age: 56; 100% f; BMI: 38.5	**i:** RYGB<br>**ii:** AGB<br>**c:** CWL	Faecal samples<br>16S rRNA gene sequencing (V3-V4) by Illumina Miseq
Lin (2018)	NRCT<br>3M	**i:** n=10; age: 37.1 (10); 60% f; BMI: 36 (4.3)<br>**c:** n=10: age: 38 (10.5); 60% f; BMI: 36 (4.6)	**i:** LSG<br>**c:** CWL	Faecal samples<br>16S rRNA gene sequencing (V4) by Illumina MiSeq
Liu R.X. (2017)	NRCT<br>3M	**i:** n=6; age: 23.3 (1.8); N.R. f; BMI: 44.5 (7.4)<br>**c:** n=25; age: 23.6 (3.8), N.R.; BMI: 20.2 (1.3)	**i:** LSG<br>**c:** ObC	Faecal samples<br>16S rRNA gene sequencing (V3-V4) by Illumina MiSeq,

				Real-time PCR
Medina (2017)	exp.<br>12M	**i:** n=5; age: N.R.; N.R. f; BMI: 37.1 (2.8)<br>**ii:** n=5; age: N.R.; N.R. f; BMI: 35.2 (2.4)<br>**c:** n=9, age: N.R., N.R. f; BMI: 38.9 (5.8)	**i:** RYGB<br>**ii:** LSG<br>**c:** CWL	Faecal samples<br>16S rRNA gene sequencing (V3-V4) by Illumina MiSeq
Murphy (2017)	RNC<br>12M	**i:** n=7; age: 48.6 (6.1); 57.1% f; BMI: 38.4 (5.2)<br>**ii:** n=7; age: 48.3 (6.1); 28.5% f; BMI: 36.9 (5.1)	**i:** RYGB<br>**ii:** LSG	Faecal samples<br>16S rRNA gene sequencing by Illumina HiSeq2000
Paganelli (2019)	RNC<br>6M	**i:** n=23; age: 44 (9.3); 91.3% f; BMI: 37.5 (4.1)<br>**ii:** n=22; age: 43.5 (12); 77.3% f; BMI: 36.6 (5.9)	**i:** RYGB<br>**ii:** LSG	Faecal samples<br>16S rRNA gene sequencing (V3-V4) by Illumina MiSeq
Pajecki (2019)	exp.<br>24M	**i:** n=9; age: 41.9; 66.7% f; BMI: 56.5	**i:** RYGB	Faecal samples<br>16S rRNA gene sequencing (V4) by Thermofisher Ion Torrent, PCR
Palleja (2016)	exp.<br>12M	**i:** n=13; age: N.R.; 61.5% f; BMI: N.R.	**i:** RYGB	Faecal samples<br>16S rRNA gene sequencing (V2) by Illumina HiSeq2000
Palmisano (2019)	RCT<br>6M	**i:** n=9; age: 44.5 (9.5); 77.8% f; BMI: 37.2 (6.9)<br>**ii:** n=16; age: 44.7 (9.4); 87.5% f; BMI: 37.2 (6.9)<br>**c:** n=25; age: 44.2 (9.3); 80% f; BMI: 22.7 (3.2)	**i:** RYGB<br>**ii:** LSG<br>**c:** HWC	Faecal samples<br>16S rRNA gene sequencing (V1-V3) by Thermofisher Ion PGM
Patrone (2016)	exp.<br>6M	**i:** n=6; age: 50.6; 81.8% f; BMI: 47.5 (7.5)	**i:** BIB	Faecal samples<br>16S rRNA gene sequencing (V4) by Illumina, real-time PCR
Rosina (1993)	Retro<br>N.R.	**i:** n=49; age: 40 (7.3); 67.4% f; BMI: N.R.	**i:** End-to-end jejunoilelal bypass<br>**ii:** End-to-side jejunoilelal bypass<br>**iii:** side-to-side jejunoilelal bypass	Samples of intestinal content: functioning jejunum, functioning ileum, middle of the excludes loop taken at reoperation.<br>Microbiological cultures
Sanmiguel (2017)	exp.<br>1M	**i:** n=8; age: 39.5 (8.7); 100% f; BMI: 44.1 (5.6)	**i:** LSG	Faecal samples<br>16S rDNA gene sequencing (V4) by Illumina HiSeq2500
Tremaroli (2015)	RCT<br>N.R.	**i:** n=7; age: 43.3 (8.1); 100% f; BMI: 42.2 (4.2)<br>**ii:** n=7; age: 50.1 (7.6); 100% f; BMI: 43 (5.1)<br>**c:** n=7; age: 35.1 (10.1); 100% f; BMI: 44.9 (4.7)	**i:** RYGB<br>**ii:** VBG<br>**c:** ObC	Faecal samples<br>Shotgun sequencing by Illumina HiSeq2000
Wang (2019)	RCT<br>3M	**i:** n=8; age: 33.3 (6.5); 100% f; BMI: N.R.<br>**ii:** n=3; age: 40.3 (6.8); 100% f; BMI: N.R.<br>**c:** n=20; N.R.	**i:** LSG<br>**i:** RYGB<br>**c:** HWC	Faecal samples<br>16S rRNA gene sequencing (V4) by Thermofisher Ion S5, real-time PCR
Zhang (2009)	NRCT<br>N.R.	**i:** n=3; age: 43.3 (8.1); 66% f; BMI: 40.6 (5.4)<br>**c:** n=3; age: 36.7 (4.0); 66% f; BMI: 22.7 (2.3)<br>**cc:** n=3; age: 35.7 (4.2); 66% f; BMI: 48.3 (7.7)	**i:** RYGB<br>**c:** HWC<br>**cc:** ObC	Faecal samples<br>16S rDNA gene sequencing (V6) and high-throughput 454, real-time PCR, PCR-DGGE
**Group 2:** Bariatric surgery and microbiota in other vertebrates				
**Author** (Year)	**Study Type/ Length**	**Sample Size and Characteristics**<br>N; Age (weeks) (SD); Sex (%), weight (g) (SD), species	**Surgery Type**	**Microbiota Samples / Analysis**
Alvarez (2018)	RCT<br>12W	n=36; age: N.R.; 0% f; weight: 250-300; rats<br>**i:** n=13 **ii:** n=13 **c:** n=10	**i:** LSG1: 1-staple load<br>**ii:** LSG2: 2-staple load<br>**c:** Sham	Intestinal content from duodenum, jejunum, ileum, caecum and faecal samples<br>16S rRNA gene sequencing (V4) by Illumina MiSeq
Basso (2016)	RCT<br>10W	n=60; age: 10; 0% f; weight: N.R.; rats	**i:** Glandular gastrectomy<br>**c:** Ctrl	Faecal samples<br>16S rRNA gene sequencing (V3-V5) by Roche GS-FLX+ Platform
Bastos (2018)	RCT<br>12W	n=17; age: N.R.; 100% f; weight: N.R.; rats<br>**i:** n=6 **c:** n=5 **cc:** n=10	**i:** Blind Loop<br>**c:** Sham<br>**cc**: Resection	Duodenum, pre- and post-anastomosis segments, ileum, cecum and faecal samples<br>Microbiological culture

Cummings (2013)	NRCT<br>18W	n=34; age: 8.; 0% f; weight: N.R.; rats<br>**i:** n=12 **c:** n=16 **cc:** n=16	**i:** Ileal interposition<br>**c:** Sham **cc:** Ctrl	Cecal samples<br>16S rRNA gene sequencing (V1-V2) by Roche GS-FLX, real-time PCR
Duboc (2017)	NRCT<br>6W	n=20; age: N.R.; 0% f; weight: 220-240; rats<br>**i:** n=5 **ii:** n=6 **c:** n=9	**i:** LSG **ii:** RYGB<br>**c:** Sham	Faecal and cecal samples<br>16S rRNA gene sequencing (V3-V4) by Illumina MiSeq
Guo (2016)	RCT<br>10W	**i:** n=10; age: 6; 0% f; weight: 291 (7.7); rats<br>**ii:** n=10; age: 6; 0% f; weight: 286 (5.8); rats<br>**c:** n=10; age: 6; 0% f; weight 303 (6.2); rats<br>**cc:** n=10; age: 6; 0% f; weight 289 (7.7); rats	**i:** RYGB **ii:** LSG<br>**c:** Sham (pair-fed)<br>**cc:** Sham (fed ad libitum)	Faecal samples<br>16S rRNA gene sequencing (V1-V3) by Roche 454 GS-FLX+ Titanium platform
Huang (2014)	RCT<br>4W	**i:** n=10; age: 20; 0% f; weight: 363 (9.3); rats<br>**c:** n=10; age: 20; 0% f; weight: 362 (25); rats<br>**cc:** n=10; age: 20; 0% f; weight: 412 (33.6); rats	**i:** LSG **c:** Sham<br>**cc:** Sham (glucose SD)	Faecal samples<br>16S rRNA gene sequencing by Sanger, PCR
Huh (2019)	RCT<br>8W	**i:** n=10 **ii:** n=10<br>**c:** n=5; age: 7; 0% f; weight: 625; rats<br>**cc:** n=5; age: 7; 0% f; weight: 796; rats<br>**ccc:** n=3	**i:** RYGB **ii:** LSG<br>**c:** Regular diet ctrl<br>**cc:** High-fat diet ctrl<br>**ccc:** Sham	Faecal samples<br>16S rRNA gene sequencing by Illumina MiSeq
Jahansouz (2017)	RCT<br>4W	n=30; age: 4; 0% f; weight: N.R.; mice<br>**i:** n=7 **ii:** n=8<br>**c:** n=7 **cc:** n=8	**i:** LSG **ii:** LSG (cohoused)<br>**c:** Sham<br>**cc:** Sham (cohoused)	Faecal samples<br>16S rRNA gene sequencing (V5-V6) by Illumina MiSeq
Jiang (2016)	NRCT<br>9W	n=N.R; age: 7; 0% f; weight: N.R.; mice	**i:** DJB **c:** Sham<br>**cc:** Sham (wild-type)	Faecal samples and different parts of the biliopancreatic limb, the roux limb, ileum, cecum, colon and rectum.<br>16S rRNA gene sequencing (V6) by Thermofisher Ion Torrent, DGGE
Kashihara (2015)	RCT<br>N.R.	n=6; age: 16; 0% f; weight: N.R.; rats<br>**i:** n=2 **c:** n=2 **cc:** n=2	**i:** DJB (diabetic)<br>**c:** Sham (diabetic)<br>**cc:** Liraglutide injection ctrl	Faecal samples<br>16S rRNA gene sequencing by Illumina MiSeq
Kim (2017)	NRCT<br>4W	n=19; age: N.R.; N.R. f; weight: N.R.; rats<br>**i:** n=4 **c:** n=7 **cc:** n=8	**i:** DES **c:** Sham<br>**cc:** Sham (pair-fed)	Cecal samples<br>16S rDNA gene sequencing (V4) by Illumina MiSeq
Li J.V. (2011)	NRCT<br>8W	**i:** n=6; age: N.R.; 0% f; weight: N.R.; rats<br>**c:** n=6; age: N.R.; 0% f; weight: N.R.; rats	**i:** RYGB<br>**c:** Sham	Faecal samples<br>16S rRNA gene sequencing (V1-V3) by PCR using a Qiagen Stool Kit
Li S. (2017)	RCT<br>3W	**i:** n=6; age: 6; 0% f; weight: 29 (5); mice<br>**ii:** n=8; age: 6; 0% f; weight: 30 (1); mice<br>**c:** n=8; age: N.R.; 0% f; weight: 30 (5); mice	**i:** DJB<br>**ii:** LSG<br>**c:** Sham	Faecal samples<br>16S rRNA gene sequencing by PCR
Liou (2013)	NRCT<br>12W	n=38; age: 22-26; 0% f; weight: N.R.; mice<br>**i:** n=14 **c:** n=11 **cc:** n=6 **ccc:** n=7	**i:** RYGB **c:** Sham<br>**cc:** Sham (wt matched)<br>**ccc:** Ctrl	Faecal samples<br>16S rRNA gene sequencing (V4) by Illumina HiSeq
Liu (2018)	NRCT<br>8W	n=11; age: 8; 0% f; weight: 180; rats<br>**i:** n=4 **c:** n=3 **cc:** n=4	**i:** RYGB<br>**c:** Sham **cc:** Ctrl	Faecal samples<br>16S rRNA gene sequencing (V4) by Illumina MiSeq
Miyachi (2017)	NRCT<br>12W	n=N.R.; age: 4-6; 0% f; weight: N.R.; rats	**i:** DJB, B-DJB, J-DJB. **c:** Sham	Faecal samples<br>16S rRNA gene sequencing by Illumina MiSeq
Mukorako (2019)	RCT<br>8W	n=21; age: N.R.; 0% f; weight: N.R.; rats<br>**i:** n=5 **ii:** n=6 **iii:** n=4 **c:** n=6	**i:** BPD/DS **ii:** LSG<br>**iii:** DS **c:** Sham	Faecal samples and intestinal content from alimentary, biliopancreatic and common limbs.<br>16S rRNA gene sequencing (V3-V4) by Illumina MiSeq
Osto (2013)	RCT<br>2W	**i:** n=8; age: N.R.; 0% f; weight: 445 (5); rats<br>**c:** n=8; age: N.R.; 0% f; weight: 435 (5); rats	**i:** RYGB<br>**c:** Sham	Intestinal content from limb, alimentary limb, common channel, cecum and colon

				Real-time PCR
Schippers (1996)	exp. 24W	**i:** n=6; age: N.R.; 0% f; weight: N.R.; dogs	**i:** RY reconstruction	Biopsy specimen from the stomach, duodenum and proximal jejunum. Microbiological investigations
Shao (2017)	RCT 9W	**i:** n=18; age: 8; 0% f; weight: N.R.; rats	**i:** RYGB, LSG	Faecal samples<br>16S rDNA gene sequencing (V4) by Illumina MiSeq
Shao (2018)	RCT 22W	n=21; age: 6; 0% f; weight: N.R.; mice<br>**i:** n=7 **c:** n=7 **cc:** n=7	**i:** LSG<br>**c:** Normal diet<br>**cc:** Sham	Faecal samples<br>16S rDNA gene sequencing (V4) by Illumina MiSeq
Wang (2019)	RCT 9W	**i:** n=N.R.; age: 12; N.R. f; weight: N.R.; rats<br>**c:** n=N.R.; age: 12; N.R. f; weight: N.R.; rats	**i:** RYGB<br>**c:** Sham	Faecal samples<br>16S rRNA gene sequencing (V4) by Illumina MiSeq
Yang (2015)	RCT 2W	**i:** n=9; age: 12; 0% f; weight: 369 (3.6); rats<br>**c:** n=9; age: 12; 0% f; weight: 369 (4.7); rats	**i:** DJB<br>**c:** Sham	Mucosa-Associated Bacteria of proximal alimentary limb, proximal biliopancreatic limb, distal common limb, and proximal colon.<br>Microbiological culture, in vitro intestinal permeability assay
Zhang (2015)	NRCT 12W	**i:** n=10; age: 8; 0% f; weight: 200; rats<br>**c:** n=10	**i:** DJB<br>**c:** Sham	Faecal samples<br>16S rRNA gene sequencing (V4) by Illumina MiSeq
**Group 3:** Probiotics and bariatric surgery				
**Author** (Year)	**Study Type/ Length**	**Sample Size and Characteristics**<br>n; Age (year) (SD); Sex %F, BMI [kg/m²] (SD)	**Surgery Type**	**Microbiota Samples / Analysis**
Chen J.C. (2016)	RNC 0.5M	n=60; age: 35.1 (8.3); 68% f; BMI: 29.2 (6.4)<br>**i:** n=20 **ii:** n=20 **c:** n=20	**i:** RYGB **ii:** Mini gastric bypass<br>**c:** Digestive enzymes	N/A
Kazzi (2018)	RCT 3M	n=40; age: 48.0 (12.7); 77.5% f; BMI: 46.2 (8)<br>**i:** n=18 **c:** n=22	**i:** LSG<br>**c:** Ctrl	N/A
Sherf-Dagan (2016)	RCT 6M	n=100; age: 41.9 (9.8); 60% f; BMI: 42.3 (4.7)<br>**i:** n=50 **c:** n=50	**i:** LSG<br>**c:** Ctrl	Faecal samples<br>16S rRNA gene sequencing (V4) by Illumina MiSeq
Woodward (2008)	RCT 6M	**i:** n=22; age: 48.6; 90.9% f; BMI: 45.7<br>**c:** n=22; age: 41.2; 84.2% f; BMI: 49.6	**i:** RYGB<br>**c:** Ctrl	N/A

AGB: Adjustable gastric banding; BIB: Bilio-intestinal bypass; BMI: Body mass index; BPD/DS: Bilopancreal diversion with duodenal switch; CWL: Conservative weight-loss; DGGE: denaturing gradient gel electrophoresis; DJB: Duodenal-jejunal bypass (B-DJB, J-DJB); DS: Duodenal switch; Exp.: Experimental study design; HWC: Healthy weight controls; LSG: Laparoscopic sleeve gastrectomy; LGB: Laparoscopic gastric bypass; ObC: Obese weight controls; NCRT: Non randomized controlled trial; N.R.: Not reported; PCR: Polymerase chain reaction; RCT: Randomized controlled trial; RNC: Randomized not controlled trial; RYGB: Roux-en-Y gastric bypass; SD: Standard deviation; SOLiD: Sequencing by oligonucleotide ligation and detection; VBG: Vertical banded gastroplasty

Text S2: Result Summaries of Group 1 and Group 2 outcomes

Overview of microbiota changes following BS in humans (Group 1)

Twenty of the twenty-nine studies reported alpha diversity (richness or biodiversity) after BS and resulted in a predominantly uniform picture. Ten studies reported an increased richness after surgery (RYGB[39,47,52,55,59,94]; LSG[46,49,50,59]; AGB[39]; DJB[41]) and six reported nil change (RYGB[37,38,54,56,76]; LSG[37,38,52,56,58]). The biodiversity was reported to increase (n=7: RYGB[42,55,59,75,94]; LSG[46,59]; DJB[41]) or remain stable (n=7: RYGB[53,54,56,76]; LSG[50,52,53,56,58]) after BS. Two studies reported lower bacterial richness and biodiversity following surgery, specifically after BIB[53] and after AGB in comparison to Conservative weight loss (CWL) control.[44]

In regards to Community Structure (beta diversity), sixteen studies reported dissimilarity following surgery in a pre-post design (RYGB[39,51,53-56,59,78,79,94]; LSG[42,50,51,53,56,58,59]; AGB[39,94]; DJB[41]; BIB[43,57]), whereas similarity was reported by 2 pre-post studies (RYGB and AGB[48]; LSG[46]). Five studies reported beta-diversity parameters for BS in comparison to a control group, reporting dissimilarity (n=4: RYGB[78,79,94]; AGB[94]; LSG[46]) and similarity (n=2: RYGB[76]; VGB[78]) in these comparisons. Wang *et al.* compared the community structure of patients following RYGB and LSG, finding dissimilarity between the groups.[59] Conversely, Tremaroli *et al.* also compared their groups and reported similarity following RYGB and VBG.[78]

In regards to changes in Firmicutes abundance after BS, twenty studies reported changes at the phylum level, with five reporting a decrease (LSG[37,38,42,58]; RYGB[45]; DJB[41]) and fourteen remained stable (RYGB[40,48,51,53-56,59]; LSG[46,49,52,53,56,59]; BIB[57]; ABG[47]). The Bacteroidetes phylum abundance was found to remain stable after BS in thirteen studies (RYGB[48,51,53-56,59]; LSG[37,38,46,49,53,56,58,59]; AGB[48]; BIB[57]), increased in four studies (LSG[42,52]; DJB[41]; RYGB[40]) and decreased in five studies (RYGB[37,38,45,52,94]; LSG[51]). Kellerer *et al.* and Palmisano *et al.* also assessed Bacteroidetes at phylum level and found higher abundance in the LSG group when compared to healthy weight controls[46] and RYGB group[56], respectively. Guitierrez-Repiso *et al.* compared the microbiota of patients following RYGB classified by their degree of postoperative weight loss, finding no significant difference at phylum level between RYGB patients.[76]

Within the Proteobacteria phylum, Gammaproteobacteria at the class level was assessed by four studies and reported consistently increased abundance after RYGB[56,94] and after RYGB when compared to controls (patients with obesity).[78,79] At species level, *E. coli* belonging to Proteobacteria increased abundance in four of four reported studies (RYGB[44,45,55]; LSG[51]) and *F. prausnitzii* bellowing to Firmicutes increased abundance in three studies (RYGB[44,48]; LSG[42]) and decreased in two studies (RYGB[45,55]). All studies which reported on Akkermansia/*Akkermansia muciniphila* of the Verrucomicrobia phylum (n=8) found a significant increase (DJB[41]; RYGB[45,48,55,56]; ABG[48]; LSG[50,51]; RYGB compared to controls (patients with obesity)[79]).

Overview of microbiota changes following BS in vertebrates other than humans (Group 2)

Twelve of the twenty-five non-human studies reported alpha diversity (richness or biodiversity) after BS. Diversity richness following BS was predominately reported as not significantly differing from sham operated controls (n=6: RYGB[84,85]; LSG[61,80,84,85]; GG[81]; DJB[93]), with only two studies reporting higher (RYGB[90]; LSG[90,92]) and one reporting lower (BPD/DS and DS[66]) than sham. Biodiversity was reported as higher (n=2: GG[81]; RYGB[85]), lower (n=3: DJB[91]; RYGB[68]; BPD/DS and DS[66]) and similar (n=5: LSG[61,80,84,92]; RYGB[65,84]) to sham groups. Community Structure (beta-diversity) was reported by twelve studies, with ten reported dissimilarity between BS and sham operated groups (LSG[84,85,90,92]; RYGB[64,65,68,84,85,90]; GG[81]; DJB[91]; BPD/DS and DS[66]). Similarity was only reported by Kim *et al.* in DES[86] and Jahansouz *et al.* and Shao *et al.* (2017) in LSG[61,68].

Overall taxonomy abundances at phylum level were assessed by twenty studies. Firmicutes phylum was reported lower following BS than sham controls by six studies (RYGB[65,69,90]; LSG[60,61,90,92]), higher in four studies (DJB[91,93]; RYGB[62,85] and not significantly different in ten[60,66,68,80,81,83,84,86,87,95]. Bacteroidetes was found to have similar abundance after BS to sham in thirteen studies (RYGB[62,68,84,85,89,90]; LSG[60,66,68,80,84,90]; GG[81]; IT[83]; DJB[87,95]; BDP/DS[66]). LSG was associated with higher abundance in Bacteroidetes compared to sham controls (LSG[60,61,92]), whereas lower abundance was found in DJB and DES groups (DJB[91,93]; DES[86]). At phylum level Verrucomicrobia abundance increased in five studies (RYGB[90]; LSG[61,90,92]; DJB[91]; DES[86]), however was also not significant in fifteen studies (RYGB[62,65,68,84,85,89]; LSG[60,61,66,68,80,84,85]; DJB[87,93,95]; GG[81]; IT[83]; BDP/DS[66]). Guo *et al.* compared the phyla of RYGB and LSG groups, finding higher Firmicutes and Proteobacteria abundance in the RYGB group compared to LSG, and higher Bacteroidetes and Actinobacteria abundance in the LSG group.[85]

Analysis at the class level showed a higher abundance in Gammaproteobacteria within the Proteobacteria phylum (n=5: GG[81]; IT[83]; DJB[95]; RYGB compared to LSG and sham groups[68,85]) and mixed outcomes for the Bacilli class within Firmicutes (Increase n=1: GG[72]; Decrease n=2: LSG[85,92]; Stable n=2: RYGB[62], DJB[95]). Twelve studies reported results at the genus level. Bifidobacterium genus within the Actinobacteria phylum was observed in higher abundance than sham operated vertebrates in five reporting studies (LSG[92]; RYGB[88]; DES[86]; DJB[87]; BDP/DS[66]). Similarly, Akkermansia genus within Verrucomicrobia was also higher in the four reporting studies (RYGB[64,91]; LSG[90,92]; DJB[91]). Within the Firmicutes phylum, Ruminococcus genus was lower following BS than sham controls in four studies (GG[81]; RYGB[84,90]; LSG[60,90]) and Clostridium in three studies (LSG[60,84]; DJB[87]).

Table S2a: Group 1 - Microbiota changes following BS in humans Results

Study	General findings	Alpha-diversity: richness	Alpha-diversity: biodiversity	Community structure/beta-diversity	Firmicutes	Bacteroidetes	Actinobacteria	Proteobacteria	Verrucomicrobia	Other	Psychological/ behavioural outcome
Aron-Wisnewsky *et al.*, 2018	RYGB resulted in more observed micriobial changes than LSG. RYGB resulted in multiple genus and species increases	↑ After RYGB and AGB in the timecourse	N.R.	RYGB: B2 (Bacteroides-Prevotella) to B1 enterotype switch; Dissimilarity between pre- and post intervention, especially for RYGB	**genus** After RYGB: ↑ *Oscillibacter spp.* ↑ *Clostridium sp.* ↑ *Roseburia spp.* ↑ *Dialister spp.* ↓ *Coprobacillus spp.* **species** ↑ *Hungatella hathewayi 1* ↓ *Anaerostipes hadrus*	**genus** After RYGB: ↑ *Alistipes shahii* ↑ *Butyricimonas* **species** After AGB and RYGB: ↑ *Butyricimonas virosa* After AGB: ↑ *Bacteroides finegoldi*	N.R.	N.R:	N.R.	**species** ↑ *F. nucleatum* after RYGB	N.R.
Bjoerneklett *et al.*, 1981	↑ Bacterial numbers in anaerobic than in aerobic cultures No significant difference between surgery groups	↔ No differences in bacterial numbers	N.R.	N.R.	N.R:	N.R.	N.R.	N.R.	N.R.	N.R.	N.R.
Campisciano *et al.*, 2018 Campisciano *et al.*, 2017	LSG resulted in a change from a Prevotella to a Bacteroides enterotype - better aligning with HWC.	↔ After bypass and LSG (Chao1)	N.R.	N.R.	**phylum** ↑ After bypass ↓ After LSG	**phylum** ↓ After bypass ↔ After LSG **genus** After bypass: ↑ *Prevotella*	**phylum** ↓ After LSG and bypass	**phylum** ↓ After LSG ↑ After bypass	**phylum** ↔ After LSG and bypass	N.R.	N.R.
Chen H. *et al.*, 2017	Increases observed for Bacteroidetes and Bifiodobacterium, and decrease in Escherichia associated with decreased inflammatory markers	N.R.	N.R.	N.R.	**phylum** ↔ After RYGB **genus** After RYGB: ↔ *Lactobacillus* ↔ *Enterocuccus*	**phylum** ↑ After RYGB	**phylum** ↔ After RYGB **genus** After RYGB: ↑ *Bifidobacterium*	**phylum** ↔ After RYGB **genus** After RYGB: ↓ *Escherichia*	**phylum** ↔ After RYGB	N.R.	N.R.
Cortez *et al.*, 2018	DJB resulted in increased microbiota richness, and increases in *Bacteroides, Akkermansia* and *Dialister.*	After DJB: ↑ At 12M (Chao1)	After DJB: ↓ At 6M ↑ At 12M (Shannon) ↑ At 6 and 12M (Simpson)	Dissimilarity between pre- and post DJB Similarity in ctrl.	**phylum** ↓ At 6M after DJB **genus** After DJB: ↑ *Dialister* ↔ *Strepococcus* ↔ *Christensen-ellaceae* ↔ *Lachnospiraceae* ↔ *Roseburia* ↔ *Faecalib-acterium* ↔ *Eubacterium*	**phylum** ↑ At 12M after DJB **genus** After DJB: ↑ *Bacteroides* ↓ *Alistipes* ↔ *Parabacteroides* ↔ *Prevotella*	**phylum** ↔ After DJB	**phylum** ↔ After DJB	**phylum** ↑ At 12M after DJB After DJB: **genus** ↑ *Akkermansia* **species** ↑ *Akkermansia muciniphila*	N.R.	N.R.

Study	General findings	Alpha-diversity: richness	Alpha-diversity: biodiversity	Community structure/beta-diversity	Firmicutes	Bacteroidetes	Actinobacteria	Proteobacteria	Verrucomicrobia	Other	Psychological/ behavioural outcome
Damms-Machado *et al.*, 2014	Bacteroidetes negatively correlated with body weight; Firmicutes positively correlated with body weight following LSG	N.R.	N.R.	Dissimilarity between pre- and post intervention, especially for LSG	**phylum**<br>↓ After LSG<br>After LSG:<br>**genus**<br>↓ *Coprococcus*<br>↓ *Dorea*<br>↓ *Ruminococcus*<br>↔ *Eubacterium*<br>↔ *Faecali-bacterium*<br>**species**<br>↑ *F. prausnitzii*	**phylum**<br>↑ After LSG<br>After LSG:<br>**genus**<br>↔ *Bacteroides*	**phylum**<br>↔ After LSG	**phylum**<br>↔ After LSG	**phylum**<br>↔ After LSG	N.R.	N.R.
Federico *et al.*, 2016	Changes in microbiota following BIB associated in beneficial changes in metabolite production	N.R.	N.R.	↓ Within similarity between pre- and post BIB	**genus**<br>After BIB:<br>↓ *Butyrivibrio*<br>↓ *Roseburia*<br>↓ *Dorea longicatena*	N.R:	N.R.	N.R.	N.R.	N.R.	N.R.
Fouladi *et al.*, 2019	Increases observed for Lactobacillales and Micrococcales after RYGB regardless of weight loss success (compared to ctrl)	N.R.	↑ After RYBG (both groups) compaired to ctrl (Shannon)	N.R.	After RYGB with successful weight loss compared to ctrl:<br>**order**<br>↑ Lactobacillales<br>**genus**<br>↑*Streptococcus*<br>↔ *Oscillibacter*<br>↔ *Lactobacillus*	N.R	After RYGB with successful weight loss compared to ctrl:<br>**order**<br>↑ Micrococcales<br>**genus**<br>↑ *Rothia*	**genus**<br>After RYGB with successful weight loss compared to ctrl:<br>↔ *Enterobacter*	After RYGB with successful weight loss compared to ctrl:<br>**order**<br>↔Verrucomicro-biales<br>**genus**<br>↔ *Akkermansia*	N.R.	N.R.
Furet *et al.*, 2010	Increases observed for Bacteroides/Prevotella and E.coli, decreases for Bifidobacterium and Lactobacillus	N.R.	N.R.	N.R.	**species**<br>After RYGB:<br>↑ *F. prausnitzii*<br>↔ *Clostridium coccoides*<br>↔ *Clostridium leptum*	**genus**<br>After RYGB:<br>↑ *Bacteroides*<br>↑ *Prevotella*	**genus**<br>After RYGB:<br>↓ *Bifidobacterium*	**species**<br>After RYGB:<br>↑ *E.coli*	N.R.	N.R.	N.R.
Graessler *et al.*, 2013	↓ Firmicutes and Bacteroidetes<br>↑ Proteobacteria, specifically *E. cancerogenus*	N.R.	N.R.	N.R.	**phylum**<br>↓ After RYGB<br>After RYGB:<br>**genus**<br>↓ *Faecalibacterium*<br>↓ *Coprococcus*<br>↓ *Anaerostipes*<br>↑ *Veillonella*<br>**species**<br>↓ *F. prausnitzii*<br>↓*Eubacterium rectale*<br>↓ *Dialister invisus*<br>↑ *Veillonella parvula*<br>↓ *Lactobacilli*<br>↓ *Clostridium spiroform*	**phylum**<br>↓ After RYGB	**phylum**<br>↓ After RYGB<br>After RYGB:<br>**genus**<br>↓ *Nakamurella*<br>**species**<br>↓ *Myobacterium kansasii*	**phylum**<br>↑ After RYGB<br>**After RYGB:**<br>**genus**<br>↓ *Helicobacter*<br>↑ *Enterobacteria*<br>↑ *Citrobacter*<br>↑ *Salmonella*<br>↑ *Shigella*<br>**species**<br>↑ *E. cancerogenus*<br>↑ *Shigella boydii*<br>↑ *Salmonella enterica*<br>↑ *Klebsiella pneumonia,*<br>↑ *E.coli*<br>↓ *C. comes*	**phylum**<br>↑ After RYGB<br>**species**<br>After RYGB:<br>↑ *Akkermansia muciniphila*	**phylum**<br>↓ Cyanobacteria<br>↑ Fusobacteria	N.R.

Study	General findings	Alpha-diversity: richness	Alpha-diversity: biodiversity	Community structure/beta-diversity	Firmicutes	Bacteroidetes	Actinobacteria	Proteobacteria	Verrucomicrobia	Other	Psychological/ behavioural outcome
Gutiérrez-Repiso *et al.*, 2019	Compared the microbiota of post-op RYGB dependent of weight loss success - finding a more diverse core microbiome with successful weight loss group	↔ No difference between groups (Chao1)	↔ No difference between groups (Shannon)	Similarity between groups according to weight status	**phylum**<br>↔ After RYGB when compared between weight loss success<br>**genus**<br>↑ *Sarcina*<br>↑ *Butyrivibrio*<br>↑ *Lachnospira*<br>↑ *Alkaliphilus*<br>after RYGB without regain compared to weight regain group	**phylum**<br>↔ After RYGB when compared between weight loss success<br>**genus**<br>↑ *5-7N15*<br>↑ *AF12*<br>after RYGB with successful weight loss compared to weight regain group	**phylum**<br>↔ After RYGB when compared between weight loss success	**phylum**<br>↔ After RYGB when compared between weight loss success<br>**genus**<br>↑ *Pseudoalteromanes*<br>after RYGB with successful weight loss compared to weight regain group	**phylum**<br>↔ After RYGB when compared between weight loss success	**phylum**<br>↔ After RYGB when compared between weight loss success<br>**genus**<br>↑ *Cetobact-erium*<br>after RYGB without regain compared to weight regain group	N.R.
Ilhan *et al.*, 2017	More substancial microbiota changes were observed post RYGB than AGB	↑ After RYGB than AGB and PreB-ctrl.	↑ After RYGB than AGB and PreB-Ob groups<br><br>↑ Equitability scores in ctrl. and RYGB than AGB and PreB-ctrl.	Dissimilarity between PreB-OP and post-surgery patients, especially RYGB	**phylum**<br>↔ After RYGB and AGB compared to ctrl<br>After RYGB compared to ctrl:<br>**class**<br>↑ Bacilli<br>**genus**<br>↑ *Veillonella*<br>↑ *Enterococcus*<br>↑ *Streptococcus*<br>After AGB compared to RYGB:<br>↑ *Holdemania*	**phylum**<br>↔ After RYGB and AGB compared to ctrl<br>After AGB compared to ctrl:<br>**class**<br>↑ Flavobacteria<br>**genus**<br>↑ *Parabacteroides*<br>After RYGB compared to ctrl:<br>↑ *Prevotella*<br>After AGB compared to RYGB:<br>↑ *Bacteroides*	**phylum**<br>↔ After RYGB and AGB compared to ctrl	**phylum**<br>↔ After RYGB and AGB compared to ctrl<br>After RYGB compared to AGB:<br>**class**<br>↑ Gammaproteo-bacteria<br>**genus**<br>↑ *Escherichia spp.*<br>After RYGB compared to ctrl:<br>↑ *Haemophilus*	**phylum**<br>↔ After RYGB and AGB compared to ctrl	**class**<br>After RYGB compared to ctrl:<br>↑ Fusobacteria	N.R.
Kellerer *et al.*, 2019	LSG results in taxomy changes in the direction of HWC.	↑ after LSG	↑ after LSG (shannon)	Dissimilarity between LSG and ctrls. Similarity between pre and post LSG.	**phylum**<br>↔After LSG<br>↓ After LSG compared to HWC<br>**genus**<br>After LSG:<br>↓ *Anaerostipes*<br>↑ *Ruminococcacae NK5A214*	**phylum**<br>↔After LSG<br>↑ After LSG compared to HWC<br>After LSG compared to HWC:<br>**order**<br>↑ Bacteroidales<br>**family**<br>↓ *Prevotellaceae*<br>After LSG:<br>↑ *Rikenellaceae*	**phylum**<br>↔After LSG	**phylum**<br>↔After LSG<br>↔ After LSG and compared to HWC	**phylum**<br>↔After LSG	N.R.	N.R.
Kong *et al.*, 2013	RYGB resulted in increases for Bacteroidetes, proteobacteria, and decreases in Firmicutes and bifiodacterium.	↑ After RYGB (Chao1, ACE)	N.R.	N.R.	**genus**<br>After RYGB:<br>↓ *Lactobacillus*<br>↓ *Dorea*<br>↓ *Blautia*<br>↑ *Peptostreptococcus*	**genus**<br>After RYGB:<br>↑ *Bacteroides*<br>↑ *Alistipes*	**genus**<br>After RYGB:<br>↓ *Bifidobacterium*	**genus**<br>After RYGB:<br>↑ *Escherichia spp.*	N.R.	N.R.	N.R.

Study	General findings	Alpha-diversity: richness	Alpha-diversity: biodiversity	Community structure/beta-diversity	Firmicutes	Bacteroidetes	Actinobacteria	Proteobacteria	Verrucomicrobia	Other	Psychological/ behavioural outcome
Lee *et al.*, 2019	Greater microbial changes were observed following RYGB than AGB, including increased F.prausnitzii following RYGB.	↓ After AGB compared to After RYGB and CWL (chao1)	↓ After AGB compared to After RYGB and CWL (PD)	Similarity between pre- and post intervention	**phylum**<br>↔ After RYGB and AGB<br>**genus**<br>After RYGB compared to AGB and CWL:<br>↑ *Faecalibacterium*	**phylum**<br>↔ After RYGB and AGB<br>**genus**<br>After RYGB and AGB:<br>↔ *Bacteroides*	**phylum**<br>↑ After RYGB<br>↔ After AGB	**phylum**<br>↑ After RYGB and AGB	**phylum**<br>↔ After RYGB and AGB<br>**genus**<br>After RYGB, AGB and CWL<br>↑ *Akkermansia*	N.R.	N.R.
Lin *et al.*, 2019	LSG resulted in increases in diversity, *Pseudobutyrivibrio* and *prevotella* when compared to lifestyle intervention weight loss.	↑ At 3M after LSG and after LSG compared to CWL (Chao1 and ACE)	N.R.	N.R.	**phylum**<br>↔After LSG<br>After LSG compared to CWL:<br>**genus**<br>↑ *Pseudobutyrivibrio*<br>**species**<br>↑ *Peptoniphilus lacrimalis*<br>↑ *Selenomonas sputigena*	**phylum**<br>↔ After LSG<br>**genus**<br>After LSG compared to CWL:<br>↑ *Prevotella sp.*	**phylum**<br>↔After LSG	**phylum**<br>↔After LSG	N.R.	N.R.	N.R.
Liu R.X. *et al.*, 2017	LSG resulted in increases in *Akkermansia muciniphila* and total gene count.	↑ Gene count After LSG	↔ After LSG (shannon)	Dissimilarity between pre- and post LSG	**genus**<br>After LSG:<br>↓ *Dorea sp.*<br>↓ *Coprococcus sp.*<br>↓ *Ruminococcus sp.*<br>↔ *Faecalibacterium sp.*	**genus**<br>After LSG:<br>↑ *Bacteroides sp.*<br>↔ *Alistipes sp.*	N.R.	N.R.	**species**<br>After LSG:<br>↑ *Akkermansia muciniphila*	N.R.	N.R.
Medina *et al.*, 2017	BS resulted in more microbial changes than CWL, with clinical changes associated with a reduction in Bacteroidetes. Specific microbiota changes differed following different surgery options.	N.R.	N.R.	Dissimilarity between pre- and post surgery, Similarity of MT	**phylum**<br>↑ After LSG<br>↔After RYGB<br>**genus**<br>After RYGB and LSG:<br>↑ *Streptococcus luteciae*<br>**species**<br>After RYGB:<br>↑ *Lactobacillales sp.*<br>↑ *Succiniclastum sp.*	**phylum**<br>↓ After LSG<br>↔After RYGB<br>**species**<br>After RYGB:<br>↑ *Bacteroides eggerthii*<br>↑ *Bacteroides coprophilus*	**phylum**<br>↑ After RGYB<br>↔After LSG	**phylum**<br>↑ After RGYB and LSG<br>**species**<br>After LSG:<br>↑ *E. coli*	**phylum**<br>↔After RYGB and LSG<br>**species**<br>After LSG:<br>↑ *Akkermansia muciniphila*	N.R.	N.R.
Murphy *et al.*, 2017	RYGB resulted in more significant microbiota changes, including increased alpha-diversity, Firmicutes and Actinobacteria. Contrasting changes were observed for Bacteroidetes.	↑ After RYGB<br>↔ After LSG	↑ After RYGB<br>↔ After LSG	N.R.	**phylum**<br>↑ After RGYB<br>↔ After LSG<br>**species**<br>After RYGB and LSG:<br>↑ *Roseburia intestinalis*	**phylum**<br>↓ after RGYB<br>↑ after LSG	**phylum**<br>↑ After RGYB<br>↔ After LSG	**phylum**<br>↔After RYGB and LSG	**phylum**<br>↔After RYGB and LSG	N.R.	N.R.

Study	General findings	Alpha-diversity: richness	Alpha-diversity: biodiversity	Community structure/beta-diversity	Firmicutes	Bacteroidetes	Actinobacteria	Proteobacteria	Verrucomicrobia	Other	Psychological/ behavioural outcome
Paganelli *et al.*, 2019	Microbial changes observed during pre-surgery diet phase. BS restored most microbial changes induced by VLCD.	N.R.	↔ After RYGB and LSG (shannon)	Dissimilarity between pre- and post surgery, Similarity between RYGB and LSG	**phylum** ↔ After RYGB and LSG **family** After RYGB and LSG: ↑ Streptococcaceae ↑ Veillonellaceae	**phylum** ↔ After RYGB and LSG	**phylum** ↓ After RGYB and LSG **family** After RYGB and LSG: ↓ Bifidobacteriaceae	**phylum** ↑ After RGYB and LSG **family** After RYGB and LSG: ↑ Enterobacteriaceae	**phylum** ↔ After RYGB and LSG	N.R.	N.R.
Pajecki *et al.*, 2019	Significant reduction in Proteobacteria was observed follwing RYGB.	↔ After RYGB (Chao1)	↔ After RYGB (shannon)	Dissimilar between pre- and post surgery for unweighted unifrac (weighted unifrac p=0.08)	**phylum** ↔ After RYGB **genus** After RYGB: ↔ *Roseburia*	**phylum** ↔ After RYGB **family** After RYGB ↔ Rikenellaceae	**phylum** ↔ After RYGB **genus** After RYGB: ↔ *Bifidobacterium*	**phylum** ↔ After RYGB **family** After RYGB ↔ Enterobacteriaceae	**phylum** ↔ After RYGB	N.R.	N.R.
Palleja *et al.*, 2016	Increased diversity observed following RYGB, correlated with observed metabolic improvements following surgery.	↑ Species richness after RYGB	↑ Gene richness: After RYGB (Shannon)	Dissimilarity between pre- and post surgery	**phylum** ↔After RYGB **genus** After RYGB: ↑ *Veillonella* ↑ *Streptococcus* **species** ↑ *E. faecalis* ↓ *F. prausnitzii*	**phylum** ↔After RYGB **genus** After RYGB: ↑ *Alistipes*	**phylum** ↔After RYGB **species** After RYGB: ↑ *Bifidobacterium dentium*	**phylum** ↑ After RYGB **species** After RYGB: ↑ *E.coli* ↑ *Klebsiella pneumoniae*	**phylum** ↔After RYGB **species** After RYGB: ↑ *Akkermansia muciniphila*	**phylum** ↑ Fusobacteria after RYGB **species** After RYGB: ↑ *F. nucleatum*	N.R.
Palmisano *et al.*, 2019	RYGB resulted in significant short and longer term changes in taxonomy. No significant changes were observed following LSG.	↔ No significant difference observed (Chaol)	↔ No significant difference observed (Shannon, simpson)	Dissimilarity between pre- and post surgery	**phylum** ↔After RYGB and LSG After RYGB to HWC: **class** ↓ Clostridia **species** ↑ *Veillonella atypica* ↑ *Veillonella dispar* ↑ *Stephrococcus gordonii* ↑ *S. australis*	**phylum** ↔After RYGB and LSG ↑ After LSG compared to RYGB	**phylum** ↔After RYGB and LSG	**phylum** ↔After LSG ↑ After RYGB at 3M After RYGB: **class** ↑ Gammaproteo-bacteria **species** ↑ *Yokenella regensburgei*	**phylum** ↔After RYGB and LSG ↑ After RYGB compared to HWC **species** After RYGB: ↑ *Akkermansia munciniphila*	**phylum** ↑ Fusobacteria After RYGB compared to HWC **species** After RYGB compared to HWC: ↑ *Fusobacter-ium varium*	↓ Preference for carbohydrates at 6M postoperative ↔ Preference for protein, vegetables and fats at 6M postoperative
Patrone *et al.*, 2016	Decreased diversity observed following BIB, with decreases in families in the Firmicutes phylum and increase in Enterobacteriaceae observed.	↓ After BIB (Chao1)	↓ At 6M after BIB (Simpson, Shannon)	Dissimilarity between pre- and post surgery	**phylum** ↔ After BIB After BIB: **family** ↓ Lachnospiraceae ↓ Clostridiaceae ↓ Ruminococcaceae ↓ Eubacteriaceae **genus** ↑ *Lactobacillus* ↑ *Megasphaera* ↑ *Acidaminococcus*	**phylum** ↔ After BIB	**phylum** ↔ After BIB **family** After BIB: ↓ Coriobacteriaceae	**phylum** ↔ After BIB **family** After BIB: ↑ Enterobacteriaceae	**phylum** ↔ After BIB	N.R.	N.R.

Study	General findings	Alpha-diversity: richness	Alpha-diversity: biodiversity	Community structure/beta-diversity	Firmicutes	Bacteroidetes	Actinobacteria	Proteobacteria	Verrucomicrobia	Other	Psychological/ behavioural outcome
Rosina *et al.*, 1993	Aerobic and anaerobic colonic microbiota observed in the ileum.	N.R.	N.R.	N.R.	N.R.	N.R.	N.R.	N.R.	N.R.	N.R.	N.R.
Sanmiguel *et al.*, 2017	LSG resulted in increased Fusobacteria and decreased Firmicutes and Bifidobacteriacaea.	↔ No significant changes after LSG	↔ No significant changes after LSG	Dissimilarity trend between pre- and post LSG	**phylum**<br>↓ After LSG<br>**genus**<br>After LSG:<br>↑ *Bulleidia*	**phylum**<br>↔ After LSG	**phylum**<br>↔ After LSG<br>After LSG:<br>**family**<br>↓ Bifidobacteriaceae<br>**genus**<br>↑ *Atopobium*	**phylum**<br>↔ After LSG	**phylum**<br>↔ After LSG	**phylum**<br>After LSG:<br>↑ Fusobacteria	YFAS score showed ↓ desire for high-calorie foods after LSG compared with baseline.<br>↓ Ratings for hedonic eating after LSG change was associated with alterations of the gut microbiome
Tremaroli *et al.*, 2015	RYGB and VBG resulted in microbiota changes, independent of BMI changes. Decreases in Firmicutes species were observed in both RYGB and VBG compared to obese controls.	N.R.	N.R.	Dissimilarity between RYGB and Obesity but not VBG ans obesity, Similarity for RYGB and VBG	**phylum**<br>↔ RYGB and VBG compared to ObC<br>**species**<br>After RYGB compared to ObC:<br>↓ *Clostridium difficile*<br>↓ *Clostridium hiranonis*<br>↓ *Gemella sanguinis*<br>After VBG compared to ObC:<br>↓ *Eubacterium rectale*<br>↓ *Roseburia intestinalis*	**phylum**<br>↔ RYGB and VBG compared to ObC	**phylum**<br>↔ RYGB and VBG compared to ObC<br>**genus**<br>After RYGB compared to VBG:<br>↓ *Bifidobacterium*	**phylum**<br>↑ After RYGB compared to ObC<br>↔ VBG compared to ObC<br>After RYGB compared to ObC:<br>**class**<br>↑ Gammaproteo-bacteria<br>**genus**<br>↑ *Escherichia*<br>↑ *Klebsiella*<br>↑ *Pseudomona*<br>**species**<br>After VBG compared to ObC:<br>↑ *E. coli*	**phylum**<br>↔ RYGB and VGB compared to ObC	N.R.	N.R.
Wang *et al.*, 2019	RYGB and LSG resulted in increases in alpha diversity and increases in genus and species in both Firmicutes and Bacteroidetes phyla.	↑ after RYGB and LSG (chao1)	↑ after RYGB and LSG (shannon)	Dissimilarity between pre- and post surgery, beta diversity↑ in patients with obesity versus ctrl. After surgery beta diversity ↓	**phylum**<br>↔ After RYGB and LSG<br>After RYGB and LSG:<br>**family**<br>↑ Streptococcaceae<br>**genus**<br>↑ *Streptococcus*<br>**species**<br>↑ *S. Salivarius*<br>↑ *S.Thermophilus*<br>After RYGB:<br>**genus**<br>↓ *Faecalibacterium*	**phylum**<br>↔ After RYGB and LSG<br>After LSG:<br>**family**<br>↑ Rikenellaceae<br>↑ Porphyromonadaceae<br>**genus**<br>↑ *Alistipes*<br>**species**<br>↑ *Alistipes Finegoldii*	**phylum**<br>↔ After RYGB and LSG	**phylum**<br>↔ After RYGB and LSG	**phylum**<br>↔ After RYGB and LSG	**phylum**<br>After RYGB and LSG:<br>↔<br>Fusobacteria	N.R.

Study	General findings	Alpha-diversity: richness	Alpha-diversity: biodiversity	Community structure/beta-diversity	Firmicutes	Bacteroidetes	Actinobacteria	Proteobacteria	Verrucomicrobia	Other	Psychological/ behavioural outcome
Zhang *et al.*, 2009	Microbiota taxonomy after RYGB differ from both HWC and ObC.	N.R.	N.R.	Dissimilarity between patients with obesity and ctrl.	**family**<br>After RYGB compared to ObC:<br>↔ Erysipelotrichaceae<br>**genus**<br>After RYGB compared to HWC:<br>↓ *Clostridia*	**family**<br>↓ Prevotellaceae After RYGB compared to ObC.	**family**<br>↔ Coriobacteriaceae in ObC	After RYGB compared to ctrls:<br>**class**<br>↑ Gammaproteo-bacteria<br>**family**<br>↔ Alcaligenaceae<br>↑ Enterobacteriacea	**genus**<br>↑ Akkermansia compared to ObC	**family**<br>After RYGB:<br>↔ Fusobacteriaceae	N.R.

AGB: Adjustable gastric banding; BIB: Bilio-intestinal bypass; ctrl: control group; CWL: Conservative weight loss; DJB: Duodenal-jejunal bypass (B-DJB, J-DJB); HWC: Healthy weight controls; LSG: Laparoscopic sleeve gastrectomy; ObC: Obese weight controls; Not reported; RYGB: Roux-en-Y gastric bypass; VBG: Vertical Banded Gastroplasty; VLCD: Very low calorie diet; YFAS: Yale food addiction scale.

Table S2b: Group 2 - Microbiota changes following BS in vertebrates other than humans Results

Study	General findings	Alpha-diversity: richness	Alpha-diversity: biodiversity	Community structure/beta-diversity	Firmicutes	Bacteroidetes	Actinobacteria	Proteobacteria	Verrucomicrobia	Other	Psychological/ behavioural outcome
Alvarez *et al.*,<br>2018	↔ No intestinal microbiome differences observed comparing LSG1, LSG2 and sham	↔ After LSG1, LSG2 and sham. (Chao1, ACE)	↔ After LSG1, LSG2 and sham. (Shannon)	N.R.	**phylum**<br>↔ After LSG1 and LSG2 compared to sham	**phylum**<br>↔ After LSG1 and LSG2 compared to sham	**phylum**<br>↑ After LSG2 compared to LSG1 and sham<br>↔ After LSG1 compared to sham	**phylum**<br>↔ After LSG1 and LSG2 compared to sham	**phylum**<br>↔ After LSG1 and LSG2 compared to sham	**phylum**<br>↔ After LSG1 and LSG2 compared to sham	N.R.
Basso *et al.*,<br>2016	Glandular gastrectomy resulted in higher gammaproteobacteria and Lactobacillus, and lower Ruminococcus compared to sham.	↔ After GG and sham (Chao1)	↑ After GG compared to sham (Shannon)	Dissimilarity between GG and ctrl.	**phylum**<br>↔ After GG compared to sham<br>**class**<br>After GG compared to sham:<br>↑ Bacilli<br>↑ Erysipelotrichia<br>↓ Clostridia<br>**genus**<br>↓ *Ruminococcus*<br>↑ *Lactobacillus*<br>↔ *Roseburia*	**phylum**<br>↔ After GG compared to sham<br>**genus**<br>After GG compared to sham:<br>↔ *Bacteroides*<br>↔ *Prevotella*	**phylum**<br>↔ After GG compared to sham<br>After GG compared to sham:<br>**class**<br>↑ Actinobacteria<br>**genus**<br>↑ *Collinsella*	**phylum**<br>↔ After GG compared to sham<br>After GG compared to sham:<br>**class**<br>↑ Gammaproteo-bacteria	**phylum**<br>↔ After GG compared to sham	N.R.	N.R.
Bastos *et al.*,<br>2018	Higher bacterial concentrations in the intestine was observed in BL and RG compared to sham	N.R.	N.R.	N.R.	N.R.	N.R.	N.R.	N.R.	N.R.	N.R.	N.R.
Cummings *et al.*,<br>2013	Phyla levels did not different between groups, however Gammaproteobacteria was observed higher in IT compared to sham or control.	N.R.	N.R.	N.R.	**phylum**<br>↔ After IT compared to sham and ctrl<br>**genus**<br>After IT compared to sham:<br>↔ *Ruminococcus*<br>↔ *Lactobacillus*<br>↔ *Roseburia*<br>↔ *Oscillibacter*	**phylum**<br>↔ After IT compared to sham and ctrl<br>**genus**<br>After IT compared to sham:<br>↔ *Bacteroides*<br>↔ *Alistipes*	**phylum**<br>↔ After IT compared to sham and ctrl	**phylum**<br>↔ After IT compared to sham and ctrl<br>After IT compared sham and ctrl:<br>**class**<br>↑ Gammaproteo-bacteria<br>**genus**<br>↑ *Escherichia*	**phylum**<br>↔ After IT compared to sham and ctrl	N.R.	N.R.
Duboc *et al.*,<br>2017	Both RYGB and LSG resulted in lower Ruminococcus than sham. LSG resulted in lower Clostridium and higher Enterobactiacae.	↔ No differences among RYGB, LSG, sham (Chao1)	↔ No changes among RYGB, LSG, sham (Simpson, Shannon)	Dissimilarity between LSG and sham, trend dissimilarity between RYGB and sham; dissimilarity between RYGB and LSG	**phylum**<br>↔ After RYGB and LSG compared to sham<br>After RYGB and LSG compared to sham:<br>**genus**<br>↓ *Ruminococcus*<br>After LSG compared to RYGB:<br>**genus**<br>↓ *Clostridium*<br>**species**<br>↓ *Clostridium perfringens*	**phylum**<br>↔ After RYGB and LSG compared to sham	**phylum**<br>↔ After RYGB and LSG compared to sham	**phylum**<br>↔ After RYGB and LSG compared to sham<br>After LSG compared to RYGB and sham:<br>**genus**<br>↑ *Enterobacteriaceae*	**phylum**<br>↔ After RYGB and LSG compared to sham	N.R.	N.R.

Study	General findings	Alpha-diversity: richness	Alpha-diversity: biodiversity	Community structure/beta-diversity	Firmicutes	Bacteroidetes	Actinobacteria	Proteobacteria	Verrucomicrobia	Other	Psychological/ behavioural outcome
Guo *et al.*, 2016	RYGB resulted in higher Proteobacteria/Gammaproteobacteria, Fusobacteria and Clostridium, whereas SG resulted higher Actinobacteria, compared with other groups.	↔ No differences among RYGB, LSG, sham (Chao1, ACE)	↑ After RYGB and LSG than sham groups (Shannon)	Dissimilarity between RYGB and LSG and sham	**phylum** ↑ After RYGB compared to LSG and sham ↓ After LSG compared to RYGB and sham **class** After LSG compared to RYGB and sham: ↓ Bacilli After RYGB and LSG compared to sham: ↑ Clostridia	**phylum** ↑ After LSG compared to RYGB and sham ↔ After RYGB compared to sham **class** After LSG compared to RYGB and sham: ↑ Bacteroidia	**phylum** ↑ After LSG compared to RYGB and sham ↔ After RYGB compared to sham	**phylum** ↑ After RYGB compared to LSG and sham ↔ After LSG compared to sham **class** After RYGB compared to LSG and sham: ↑ Betaproteobacteria ↑ Gammaproteo-bacteria	**phylum** ↔ After RYGB and LSG compared to sham	**genus** After RYGB compared to LSG and sham ↑ *Fusobacteria*	N.R.
Huang *et al.*, 2014	Microbial changes in both diabetic and non-diabetic rats after LSG. Nil phylum changes observed in sham.	N.R.	N.R.	N.R.	**phylum** ↓ After LSG ↔ After LSG compared to sham **genus** After LSG: ↓ *Clostridium* ↓ *Ruminococcus*	**phylum** ↑ After LSG ↔ After LSG compared to sham **genus** After LSG: ↑ *Prevotella* ↑ *Parabacteroides*	**phylum** ↔ After LSG compared to sham	**phylum** ↔ After LSG compared to sham	**phylum** ↔ After LSG compared to sham	N.R.	N.R.
Huh et al., 2019	Both RYGB and LSG resulted in decreases in Firmicutes and increases in Proteobacteria and Verrucomicrobia. Sham resulted in decreased diveristy in microbiota.	↑After RYGB and LSG ↑ After RYGB and LSG compared to sham (chao1)	N.R.	Dissimilarity between groups at week 1. RYGB and LSG groups remained dissimilar to sham at 4 and 8 weeks after surgery	**phylum** ↓ After RYGB and LSG and compared to sham **genus** After RYGB and LSG: ↓ *Lactococcus* ↓ *Dorea* ↓ *Ruminococcus* ↓ *Clostridiales* ↓ *Ruminococcaceae*	**phylum** ↔ After RYGB and LSG compared to sham **genus** After RYGB and LSG: ↑ *Prevotella* After LSG: ↑ *Bacteroides* After RYGB: ↓ *Bacteroides*	**phylum** ↔ After RYGB and LSG compared to sham **genus** After LSG: ↑ *Yaniella* After RYGB: ↑ *Eggerthella*	**phylum** ↑ After RYGB and LSG and compared to sham **genus** After RYGB and LSG: ↑ *Sutterella* ↑ *Enterobacteriaceae* After LSG: ↑ *Psychrobacter*	**phylum** ↑ After RYGB and LSG compared to sham **genus** After RYGB and LSG: ↑ *Akkermansia*	N.R.	N.R.
Jahansouz *et al.*, 2017	LSG resulted in decrease in Firmicutes and increase in Bacteroidetes (both compared to pre-op and sham group).	↔ No differences after LSG or compared to sham (ACE)	↔ No differences after LSG or compared to sham (Shannon)	Similarity between individually housed LSG and sham	**phylum** ↓ After LSG ↓ After LSG compared to sham (for comparisons of co-housed and individually housed)	**phylum** ↑ After LSG ↑ After LSG compared to sham (for comparisons of co-housed and individually housed)	**phylum** ↔ After LSG compared to sham for comparison of individually housed) ↓ After LSG compared to sham for comparison of cohoused housed)	**phylum** ↔ After LSG compared to sham (for comparisons of co-housed and individually housed)	**phylum** ↔ After LSG compared to sham (for comparisons of co-housed and individually housed)	N.R.	↔ Cohousing did not affect metabolic outcomes of LSG or sham.
Jiang *et al.*, 2016	DJB resulted in ↑ Firmicutes and ↓ Actinobacteria and Proteobacteria compared to sham. These were correlated with reduced inflammation.	N.R.	↓ After DJB compared to sham	Dissimilarity between DJB and sham	**phylum** ↑ After DJB compared to sham **class** After DJB: ↓ Clostridiales	**phylum** ↓ After DJB compared to sham	**phylum** ↓ After DJB compared to sham	**phylum** ↓ After DJB compared to sham	**phylum** ↑ After DJB compared to sham After DJB compared to sham: **family** ↑ Verrucomicro-biaceae **genus** ↑ *Akkermansia*	N.R.	N.R.

Study	General findings	Alpha-diversity: richness	Alpha-diversity: biodiversity	Community structure/beta-diversity	Firmicutes	Bacteroidetes	Actinobacteria	Proteobacteria	Verrucomicrobia	Other	Psychological/ behavioural outcome
Kashihara *et al.*, 2015	DJB resulted in lower Bacteroidia and higher Gammaproteobacteria than sham and ctrl.	N.R.	N.R.	N.R.	**phylum**<br>↔ After DJB compared to sham<br>**class**<br>After DJB compared to sham and ctrl:<br>↔ Bacilli<br>↔ Clostridia	**phylum**<br>↔ After DJB compared to sham<br>**class**<br>After DJB compared to sham and ctrl:<br>↓ Bacteroidia	**phylum**<br>↔ After DJB compared to sham	**phylum**<br>↔ After DJB compared to sham<br>**class**<br>After DJB compared to sham and ctrl:<br>↑ Gammaproteo-bacteria	**phylum**<br>↔ After DJB compared to sham	N.R.	N.R.
Kim *et al.*, 2017	DES resulted in higher *Akkermansia muciniphila* and Bifidobacteria than sham. Nil significant difference between DES and sham for Firmicutes phylum and lower Bacteroidetes phlyum in DES was observed.	N.R.	N.R.	Similarity in clustering of DES and ctrl groups	**phylum**<br>↔ After DES compared to sham<br>After DES compared to sham:<br>**class**<br>↑Erysipelotrichi<br>**genus**<br>↓ *Lactobacillus*<br>↓ *Streptokokken*<br>↑ *Enterococcus*<br>↑ *Allobaculum*<br>**species**<br>↑ *C. perfringens*	**phylum**<br>↓ After DES compared to sham<br>After DES compared to sham:<br>**class**<br>↓ Bacteroidia<br>**genus**<br>↓ *Prevotella*<br>↑ *Parabacteroides*	**phylum**<br>↔ After DES compared to sham<br>**genus**<br>After DES compared to sham:<br>↓ *Rothia*<br>↑ *Bifidobacterium*	**phylum**<br>↔ After DES compared to sham	**phylum**<br>↑ after DES compared to sham<br>**species**<br>After DES compared to sham:<br>↑ *Akkermansia muciniphila*	After DES compared to sham:<br>**class**<br>↑ Mollicutes	N.R.
Li J.V. *et al.*, 2011	Shift toward Gammaproteobacteria after RYGB particularly *Enterobacter hormaechei* compared with ctrl.<br>Strong correlations between relative suspension growth (RSG) and the Enterobacteriaceae and Pasteurellaceae	N.R.	N.R.	N.R.	**phylum**<br>↑ after RYGB compared to sham<br>After RYGB compared to sham:<br>**class**<br>↔ Bacilli<br>↓ Clostridia	**phylum**<br>↔ After RYGB compared to sham	**phylum**<br>↔ After RYGB compared to sham	**phylum**<br>↑ after RYGB compared to sham<br>After RYGB compared to sham:<br>**class**<br>↑ Gammaproteo-bacteria	**phylum**<br>↔ After RYGB compared to sham	N.R.	N.R.
Li S. *et al.*, 2017	↓ Intestinal inflammation after DJB in DSS-induced colitis.	N.R.	N.R.	N.R.	**family**<br>After DJB and LSG compared to sham:<br>↑ Lactobacillales	N.R.	N.R.	N.R.	N.R.	N.R.	N.R.
Liou *et al.*, 2013	RYGB resulted in higher Enterobacteriales and Verrucomicrobiales compared to sham.<br>↑ Archeae After RYGB	N.R.	N.R.	Dissimilarity between pre- and post RYGB.<br>Dissimilarity between RYGB and sham/food restricted weight loss	**phylum**<br>↓ After RYGB compared to sham<br>After RYGB compared to sham:<br>**order**<br>↑ Clostridiales<br>**genus**<br>↑ *Clostridium*<br>↓ *Lactobacillus*	**phylum**<br>↑ After RYGB compared to sham<br>After RYGB compared to sham:<br>**order**<br>↔ Bacteroidales<br>**genus**<br>↑ *Alistipes*	**phylum**<br>↔ After RYGB compared to sham	**phylum**<br>↑ After RYGB compared to sham<br>**order**<br>After RYGB compared to sham:<br>↑ Enterobacteriales	**phylum**<br>↑ After RYGB compared to sham<br>After RYGB compared to sham:<br>**order**<br>↑ Verrucomicrobiales<br>**genus**<br>↑ *Akkermansia*	N.R.	N.R.

Study	General findings	Alpha-diversity: richness	Alpha-diversity: biodiversity	Community structure/beta-diversity	Firmicutes	Bacteroidetes	Actinobacteria	Proteobacteria	Verrucomicrobia	Other	Psychological/ behavioural outcome
Liu *et al.,* 2018	RYGB resulted in increased Bacteroidetes, Actinobacteria, Proteobacteria and Fusobacteria, with decreased Firmicutes.	N.R.	↔ After RYGB compared to sham	Dissimilarity between RYGB and sham	**phylum** ↓ After RYGB and when compared to sham **order** After RYGB compared to sham: ↑ Lactobacillales ↓ Clostridiales	**phylum** ↑ After RYGB and when compared to sham	**phylum** ↑ After RYGB ↔ After RYGB compared to sham	**phylum** ↔ After RYGB compared to sham **order** After RYGB compared to sham: ↑ Burkholderiales ↑ Enterobacteriales	**phylum** ↔ After RYGB compared to sham **order** ↓ Verrucomicrobia after RYGB compared to sham	**phylum** After RYGB: ↑ Fusobacteria	N.R.
Miyachi *et al.,* 2017	B-DJB resulted in higher Bifidobacterium and lower clostridium than other methods.	N.R.	N.R.	N.R.	**phylum** ↔ After DJB compared to sham **genus** After B-DJB compared to J-DJB and sham: ↓ *Clostridium* ↓ *Turicibacter* ↔ *Lactobacillus*	**phylum** ↔ After DJB compared to sham **genus** After B-DJB compared to J-DJB and sham: ↓ *Bacteroides*	**phylum** ↔ After DJB compared to sham **genus** After B-DJB compared to J-DJB and sham: ↑ *Bifidobacterium* ↑ *Olsenella*	**phylum** ↔ After DJB compared to sham	**phylum** ↔ After DJB compared to sham	N.R.	N.R.
Mukorako *et al.,* 2019	↑ *Bifidobacterium* and ↓ *Peptostreptococca ceae* and *Clostridiaccae* in common and alimentary limbs After BPD/DS and DS	↓ After DS compared to sham ↔ After BPD/DS and LSG compared to sham (Chao1)	↓ After BPD/DS and DS compared to sham ↔ After LSG compared to sham (shannon)	Dissimilarity between BPD/DS and DS compared to sham	**phylum** ↔ After BDP/DS, DS and LSG compared to sham After BDP/DS and DS compared to sham: **class** ↓ *Clostridiales* **family** ↓ *Peptostrepto-coccaceae* ↓ *Clostridiaceae*	**phylum** ↔ After BDP/DS, DS and LSG compared to sham **order** After BDP/DS and DS compared to sham: ↓ *Bacteroidales*	**phylum** ↔ After BDP/DS, DS and LSG compared to sham After BDP/DS and DS compared to sham: **order** ↑ *Bifidobacteriales* **genus** ↑ *Bifidobacterium*	**phylum** ↔ After BDP/DS, DS and LSG compared to sham	**phylum** ↔ After BDP/DS, DS and LSG compared to sham	N.R.	N.R.
Osto *et al.,* 2013	Most substantial shifts in the composition of the microbiota in the alimentary limb and the common channel.	N.R.	N.R.	N.R.	**genus** After RYGB compared to sham: ↓ *Lactobacillus spp.*	**genus** After RYGB compared to sham: ↑ *Prevotella spp.* (common channel, the alimentary limb and in the colon)	**genus** After RYGB compared to sham: ↑ *Bifidobacterium spp.* (common channel, the alimentary limb and in the colon)	N.R.	N.R.	N.R	N.R.
Schippers *et al.,* 1996	↑ organisms in the stomach, Roux limb, jejunum and duodenum; the highest numbers near the gastro-jejunostomy ↑ Numbers in the bacterial microbiota similar in aerobic and anaerobic bacteria	N.R.	N.R.	N.R.	N.R.	N.R.	N.R.	N.R.	N.R.	N.R.	N.R.

Study	General findings	Alpha-diversity: richness	Alpha-diversity: biodiversity	Community structure/beta-diversity	Firmicutes	Bacteroidetes	Actinobacteria	Proteobacteria	Verrucomicrobia	Other	Psychological/ behavioural outcome
Shao *et al.*, 2017	RYGB resulted in more substancial and persistent microbial changes than LSG, including increased Proteobacteria, Gammproteobacteria and Bacteroidaceae.	N.R.	↓ After RYGB and compared to LSG and sham (Shannon)	Similarity between LSG and sham; Dissimilarity between RYGB and sham.	**phylum** ↔ After RYGB and LSG compared to sham After RYGB compared to LSG and sham: **order** ↑ Lactobacillales ↑ Erysipelotrichales ↓ Clostridiales	**phylum** ↔ After RYGB and LSG compared to sham **family** After RYGB: ↑ Bacteroidaceae	**phylum** ↔ After RYGB and LSG compared to sham	**phylum** ↑ After RYGB compared to sham ↔ After LSG compared to sham **class** After RYGB compared to LSG and sham: ↑ Gammaproteo-bacteria **order** After LSG: ↑ Desulfo-vibrionaceae	**phylum** ↔ After RYGB and LSG compared to sham	**phylum** After LSG: ↑ Cyanobacteria	N.R.
Shao *et al.*, 2018	↑ Diminished diurnal oscillation of gut microbiota after LSG ↑ Richness of gut microbiota after LSG	↑ After LSG compared to sham (Chao1)	↔ No difference between LSG and sham (Shannon)	Dissimilarity between LSG and sham	**phylum** ↓ After LSG compared to sham **class** After LSG: ↓ Erysipelotrichia ↓ Bacilli	**phylum** ↑ After LSG compared to sham **genus** After LSG: ↑ *Bacteroides* ↑ *Parabacteroides*	**phylum** ↔ After LSG compared to sham **genus** After LSG: ↑ *Bifidobacterium*	**phylum** ↔ After LSG compared to sham	**phylum** ↑ After LSG compared to sham **genus** After LSG: ↑ *Akkermansia*	N.R.	N.R.
Wang *et al.*, 2019	RYGB observed to reduce inflammation, and resulted in lower Firmicutes and higher Proteobacteria compared to sham.	N.R.	N.R.	N.R.	**phylum** ↓ After RYGB compared to sham After RYGB compared to sham: **order** ↔ Erysipelotrichales ↓ Lactobacillales **family** ↓ Clostridiaceae	**phylum** ↔ After RYGB compared to sham	**phylum** ↔ After RYGB compared to sham	**phylum** ↑ After RYGB compared to sham **order** After RYGB compared to sham: ↑ Enterobacteriales	**phylum** ↔ After RYGB compared to sham	N.R.	N.R.
Yang *et al.*, 2016	DJB altered microbiota, reduced intestinal permeability and induced mucosal hypertorphy. ↑ bacterial numbers in alimentay and common limb after DJB compared to sham.	N.R.	N.R.	N.R.	N.R.	N.R.	N.R.	N.R.	N.R.	N.R.	N.R.
Zhang X. *et al.*, 2016	DJB resulted in ↓ Bacteroidia and ↑ Firmicutes and Proteobacteria compared to sham.	↔ After DJB and sham (Chao1, ACE)	N.R.	N.R.	**phylum** ↑ After DJB compared to sham	**phylum** ↓ After DJB compared to sham	**phylum** ↔ After DJB compared to sham	**phylum** ↑ After DJB compared to sham	**phylum** ↔ After DJB compared to sham	N.R.	N.R.

BL: Blind loop; BPD/DS: Bilopancreal diversion with duodenal switch; ctrl: Control group; DES: Duodenal endoluminal barrier sleeve; DJB: Duodenal-jejunal bypass (B-DJB, J-DJB); DS: Duodenal switch; GG: Glandular gastrectomy; IT: Ileal Interposition; LSG: Laparoscopic sleeve gastrectomy; N.R.: Not reported; RG: resection group; RYGB: Roux-en-Y gastric bypass

Table S3: Group 3 – Bariatric surgery and probiotics

Study	Sample size & characterization	Study type, length, follow-up	Type of surgery	Probiotic: application, dosage [CFU/day]	Methods	Outcomes	Microbiota outcomes
Chen J.C. *et al.*, 2016	Total n=60 (f/m=41/19), Mean age 35.1 ± 8.3 years, Mean BMI 29.2 ± 6.4 kg/m² PB1: n=20 PB2: n=20 Digestive enzymes group C: n=20; *Taiwan*	RNCT 14 days Follow-up: no	(i) RYGB (ii) Mini gastric bypass	PB1: 1 g Clostridium butyrium MIYAIRI ($5 * 10^9$) PB2: 300 mg Bifidobacterium longum BB536 ($8 * 10^9$) Ctrl: Digestive enzymes (Aczym, containing 100 mg takadiastase N, 20 mg cellulase AP, 50 mg lipase MY, 100 mg pancreatin) Twice daily (Application N.R.)	Quality of life: modified Gastrointestinal Quality of Life Index (mGIQLI) at baseline and at 14 days postoperative	↑ mGIQLI after the 2-week intervention in all 3 groups regardless of operative procedure and prescriptions ↑ Improvement of complaints of abdominal pain, abdominal bloating, excessive passage of gas, foul smell of flatulence, belching, abdominal noises, and heartburn in the entire sample; Probiotics and digestive enzymes had similar efficacy. post BMI: N.R.	N.R.
Kazzi *et al.*, 2018	Total n=40 (f/m=31/9), Mean age 48.0 ± 12.7 years Mean BMI 46.2 ± 8.0 kg/m² PB: n=18 Ctrl: n=22; *USA*	RCT 3M Follow-up: 6 weeks	LSG	PB: LactoWise®: $4.5 * 10^9$ of Bacillus coagulans and galactomannans (300 mg); Ctrl: 600 mg of calcium citrate yielding 126 mg of elemental calcium Once daily (capsule)	Quality of life: Gastrointestinal Quality of Life Index (GIQLI) at baseline and at 2 and 6 weeks and at 3M postoperative	↑ GIQLI over time in PB and ctrl. ↔ No group differences regarding the degree of improvement in the GIQLI post BMI: N.R.	N.R.
Sherf-Dagan *et al.*, 2016	Total n=100 (f/m=60/40), Mean age 41.9 ± 9.8 years, Mean BMI 42.3 ± 4.7 kg/m² Morbidly obese non-alcoholic fatty liver disease (NAFLD), PB: n=50 Ctrl: n=50; *Israel*	RCT 6M Follow-up: 6M	LSG	PB: Bio-25;Supherb: 1) Lactobacillus acidophilus 2) Bifidobacterium bifidum 3) Lactobacillus rhamnosus 4) Lactococcus lactis 5) Lactobacillus casei 6) Bifidobacterium breve 7) Streptococcus thermophiles 8) Bifidobacterium longum 9) Lactobacillus paracasei 10) Lactobacillus plantarum 11) Bifidobacterium infatis $>25 * 10^9$ Ctrl: Placebo Twice daily (capsule)	Liver associated outcomes: Abdominal-ultrasound (HRI score) Biochemical parameters: uniform laboratory methods Fibrosis associated outcomes: Shear-wave elastography Quality of life (QoL): SF-12 test Microbiota composition: faecal samples at baseline, 6M, 12M and stored at -80 °C; 16S rRNA sequencing (Illumina MiSeq) at baseline, and at 3, 6, 12M	**Primary outcome** ↓ In liver fat content and NAFLD remission in PB and ctrl **Secondary outcome** ↓ Fibrosis, liver-enzymes, C-reactive protein (CRP), leptin and cytokeratin-18 levels in PB and ctrl ↑ Biochemical metabolic parameters glucose, haemoglobin A1c, Homeostasis Model Assessment, total cholesterol and triglycerides ↑ QOL in PB and ctrl at 6M ↔ No significant difference between PB and ctrl regarding BMI and waist circumference, ↔ No group differences regarding the degree of improvement in the mGIQLI score	↔ Alpha diversity at each timepoint between PB and ctrl ↔ No significant difference between PB and ctrl regarding microbiota composition, except at 6M for ↑ Proteobacteria, Actinobacteria, *Collinsella* genus in PB - All N.S. at 12M ↑ Ratio of Firmicutes/Bacteroidetes in PB and ctrl
Woodard *et al.*, 2008	Total n=44 PB: n=22 (f=90.9%), Mean age 48.6 years, Mean BMI 45.7 kg/m² Ctrl: n=22 (f=84.2%), Mean age 41.2 years Mean BMI 49.6 kg/m² *USA*	RCT 6M Follow-up: no	RYGB	Pb: Puritan's Pride®: Lactobacillus $2.4 * 10^9$ Ctrl: N.R. Once daily (capsule)	Quality of life: Gastrointestinal-related quality-of-life index (GIQOL), BO measurement was determined by hydrogen (H2) breath testing using the HBT Sleuth® at baseline and at 3, 6M	↓ H2 measures in PB ↑ Excess weight loss in PB than in ctrl at 6 week and 3M, ↑ Vitamin B12 levels in PB ↑ GIQOL in PB and ctrl at all time points ↔ No group differences regarding the degree of improvement in the GIQOL score	↓ BO at 6M in PB

BMI: Body mass index, BO: Bacterial overgrowth, CFU: Colony forming unit; ctrl: Control group f/m: female/male; GIQLI: Gastrointestinal-related Quality of Life;H2: hydrogen, LSG: Laparoscopic sleeve gastrectomy; mGIQLI: modified Gastrointestinal Quality of Life index; NAFLD: Non-alcoholic fatty liver disease, N.R.: Not reported; PB: Probiotic group, QoL: Quality of life; RYGB: Roux-en-Y gastric bypass, SF-12: Short-Form health survey

Table S4a: Subgroup 1 – Pre-post comparisons in Humans

Study	Alpha-diversity: richness	Alpha-diversity: biodiveristy	Community Structure	Firmicutes	Bacteroidetes	Actinobacteria	Proteobacteria	Verrucomicrobia	Other
Campisciano *et al.*, 2018<br>Campisciano *et al.*, 2017	Chao1<br>Bypass: ↔<br>LSG: ↔	N.R.	N.R.	After LSG:<br>**phylum:** ↓ Firmicutes<br>After bypass:<br>**phylum:** ↑ Firmicutes	After LSG:<br>**phylum:** ↔ Bacteroidetes<br>After bypass:<br>**phylum:** ↓ Bacteroidetes<br>**genus:** ↑ *Prevotella*	After LSG:<br>**phylum:** ↓ Actinobacteria<br>After bypass:<br>**phylum:** ↓ Actinobacteria	After LSG:<br>**phylum:** ↓ Proteobacteria<br>After bypass:<br>**phylum:** ↑ Proteobacteria	After LSG and bypass:<br>**phylum:** ↔ Verrucomicrobia	N.R.
Chen H. *et al.*, 2017	N.R.	N.R.	N.R.	After RYGB:<br>**phylum:** ↔ Firmicutes<br>**genus:** ↔ *Lactobacillus, Enterocuccus*	After RYGB:<br>**phylum:** ↑ Bacteroidetes	After RYGB:<br>**phylum:** ↔ Actinobacteria<br>**genus:** ↑ *Bifidobacterium*	After RYGB:<br>**phylum:** ↔ Proteobacteria<br>**genus:** ↓ *Escherichia*	After RYGB:<br>**phylum:** ↔ Verrucomicrobia	N.R.
Cortez *et al.*, 2018	Chao1<br>DJB: ↑ at 12M	Shannon, Simpson<br>DJB: ↑ at 12M	Dissimilarity	After DJB:<br>**phylum:** ↓ Firmicutes<br>**genus:** ↑ *Dialister,* ↔ *Strepococcus, Christensenellaceae, Lachnospiraceae, Roseburia, Faecalibacterium, Eubacterium*	After DJB:<br>**phylum:** ↑ Bacteroidetes<br>**genus:** ↑ *Bacteroides,* ↓ *Alistipes,* ↔ *Parabacteriodes, Prevotella*	After DJB:<br>**phylum:** ↔ Actinobacteria	After DJB:<br>**phylum:** ↔ Proteobacteria	After DJB:<br>**phylum:** ↑ Verrumicrobia<br>**genus:** ↑ *Akkermansia*<br>**species:** ↑ *Akkermansia muciniphila*	N.R.
Damms-Machado *et al.*, 2014	N.R.	N.R.	Dissimilarity	After LSG:<br>**phylum:** ↓ Firmicutes<br>**genus:** ↓ *Coprococcus, Dorea, Ruminococcus,* ↔ *Eubacterium, Faecalibacterium*<br>**species:** ↑ *F. prausnitzii*	After LSG:<br>**phylum:** ↑ Bacteroidetes<br>**genus:** ↔ *Bacteroides*	After LSG:<br>**phylum:** ↔ Actinobacteria	After LSG:<br>**phylum:** ↔ Proteobacteria	After LSG:<br>**phylum:** ↔ Verrucomicrobia	N.R.
Graessler *et al.*, 2013	N.R.	N.R.	N.R.	After RYGB:<br>**phylum:** ↓ Firmicutes<br>**genus:** ↓ *Faecalibacterium, Coprococcus, Anaerostipes,* ↑ *Veillonella*<br>**species:** ↓ *F. prausnitzii, Lactobacilli, Eubacterium rectale, Dialister invisus, Clostridium spiroform,* ↑ *Veillonella parvula*	After RYGB:<br>**phylum:** ↓ Bacteroidetes	After RYGB:<br>**phylum:** ↓ Actinobacteria<br>**genus:** ↓ *Nakamurella*<br>**species:** ↓ *Myobacterium kanasii*	After RYGB:<br>**phylum:** ↑ Proteobacteria<br>**genus:** ↑ *Enterobacteria, Citrobacteria, Salmonella, Shigella,* ↓ *Heliobacter*<br>**species:** ↑ *E. ancerogenus, Shigella boydii, Salmonella enterica, Klebsiella pneumonia, E.coli,* ↓ *C. comes*	After RYGB:<br>**phylum:** ↑ Verrucomicrobia<br>**species:** ↑ *Akkermansia muciniphila*	After RYGB:<br>**phylum:** ↓ Cyanobacteria, ↑ Fusobacteria<br>**species:** ↓ *Treponema pallidum, Brachyspira hyodysenteriae, Fusobacterium periodonticum*
Kellerer *et al.*, 2019	LSG: ↑	Shannon<br>LSG: ↑	Similarity	After LSG:<br>**phylum:** ↔ Firmicutes<br>**genus:** ↑ *Ruminococcaceae NK5A214,* ↓ *Anaerostripes*	After LSG:<br>**phylum:** ↔ Bacteroidetes<br>**family:** ↑ Rikenellaceae	After LSG:<br>**phylum:** ↔ Actinobacteria	After LSG:<br>**phylum:** ↔ Proteobacteria	After LSG:<br>**phylum:** ↔ Verrucomicrobia	N.R.
Kong *et al.*, 2013	Chao1, ACE<br>RYGB: ↑	N.R.	N.R.	After RYGB:<br>**genus:** ↓ *Lactobacillus, Dorea, Blautia,* ↑ *Peptostreptococcus*	After RYGB:<br>**genus:** ↑ *Bacteroides, Alistripes*	After RYGB:<br>**genus:** ↓ *Bifidobacterium*	After RYGB:<br>**genus:** ↑ *Escherichia*	N.R.	N.R.
Lee *et al.*, 2019	N.R.	N.R.	Similarity	After RYGB and AGB:<br>**phylum:** ↔ Firmicutes<br>After RYGB:<br>**genus:** ↑ *Faecalibacterium*<br>After AGB:<br>**genus:** ↓ *Faecalibacterium*	After RYGB and AGB:<br>**phylum:** ↔ Bacteroidetes<br>**genus:** ↔ Bacteroides	After RYGB:<br>**phylum:** ↑ Actinobacteria<br>After AGB:<br>**phylum:** ↔ Actinobacteria	After RYGB and AGB:<br>**phylum:** ↑ Proteobacteria	After RYGB and AGB:<br>**phylum:** ↔ Verrucomicrobia<br>**genus:** ↑ *Akkermansia*	N.R.
Lin *et al.*, 2019	Chao1, ACE<br>LSG: ↑	N.R.	N.R.	After LSG:<br>**phylum:** ↔ Firmicutes	After LSG:<br>**phylum:** ↔ Bacteroidetes	After LSG:<br>**phylum:** ↔ Actinobacteria	After LSG:<br>**phylum:** ↔ Proteobacteria	After LSG:<br>**phylum:** ↔ Verrucomicrobia	N.R.

Study	Alpha-diversity: richness	Alpha-diversity: biodiveristy	Community Structure	Firmicutes	Bacteroidetes	Actinobacteria	Proteobacteria	Verrucomicrobia	Other
Liu R.X. *et al.*, 2017	LSG: ↑ at 3M	N.R.	Dissimilarity	After LSG:<br>**genus:** ↓ *Dorea sp., Coprococcus sp., Ruminococcus sp.,* ↔ *Faecalibacterium sp.*	After LSG:<br>**genus:** ↑ *Bacteroides sp.,* ↔ *Alistipes sp.*	N.R.	N.R.	After LSG:<br>**species:** ↑ *Akkermansia muciniphila*	N.R.
Medina *et al.*, 2017	N.R.	N.R.	Dissimilarity	After LSG:<br>**phylum:** ↑ Firmicutes<br>After LSG and RYGB:<br>**genus:** ↑ *Streptococcus luteciae,* ↓ *Lactobacilliales*<br>After RYGB:<br>**phylum:** ↔ Firmicutes<br>**species:** ↑ *Succiniclastum*	After LSG:<br>**phylum:** ↓ Bacteroidetes<br>After RYGB:<br>**phylum:** ↔ Bacteroidetes<br>**species:** ↑ *Bacteroides eggerthii, Bacteroides coprophilus*	After RYGB:<br>**phylum:** ↑ Actinobacteria<br>After LSG:<br>**phylum:** ↔ Actinobacteria	After RYGB and LSG:<br>**phylum:** ↑ Proteobacteria<br>After LSG:<br>**species:** ↑ *E. coli*	After RYGB and LSG:<br>**phylum:** ↔ Verrucomicrobia<br>After LSG:<br>**species:** ↑ *Akkermansia muciniphila*	N.R.
Murphy *et al.*, 2017	RYGB: ↑<br>LSG: ↔	RYGB: ↑<br>LSG: ↔	N.R.	After RYGB:<br>**phylum:** ↑ Firmicutes<br>After LSG:<br>**phylum:** ↔ Firmicutes<br>After RYGB and LSG:<br>**species:** ↑ *Roseburia intestinalis*	After RYGB:<br>**phylum:** ↓ Bacteroidetes<br>After LSG:<br>**phylum:** ↑ Bacteroidetes	After RYGB:<br>**phylum:** ↑ Actinobacteria<br>After LSG:<br>**phylum:** ↔ Actinobacteria	After RYGB and LSG:<br>**phylum:** ↔ Proteobacteria	After RYGB and LSG:<br>**phylum:** ↔ Verrucomicrobia	N.R.
Paganelli *et al.*, 2019	N.R.	Shannon<br>RYGB: ↔<br>LSG: ↔	Dissimilarity	After RYGB and LSG:<br>**phylum:** ↔ Firmicutes<br>**family:** ↑ Stephococcaceae, Veillonaceae	After RYGB and LSG:<br>**phylum:** ↔ Bacteroidetes	After RYGB and LSG:<br>**phylum:** ↓ Actinobacteria<br>**family:** ↓ Bifidobacteriaceae	After RYGB and LSG:<br>**phylum:** ↑ Proteobacteria<br>**family:** ↑ Enterobacteriaceae	After RYGB and LSG:<br>**phylum:** ↔ Verrucomicrobia	N.R.
Pajecki *et al.*, 2019	Chao1<br>RYGB: ↔	Shannon<br>RYGB: ↔	Dissimilarity (unweighted)	After RYGB:<br>**phylum:** ↔ Firmicutes<br>**genus:** ↔ *Roseburia*	After RYGB:<br>**phylum:** ↔ Bacteroidetes<br>**family:** ↔ Rikenellaceae	After RYGB:<br>**phylum**: ↔ Actinobacteria<br>**genus:** ↔ *Bifidobacterium*	After RYGB:<br>**phylum:** ↓ Proteobacteria<br>**family:** ↔ Enterobacteriaceae	After RYGB:<br>**phylum:** ↔ Verrucomicrobia	N.R.
Palleja *et al.*, 2016	RYGB: ↑	Shannon<br>RYGB: ↑ at 12M	Dissimilarity	After RYGB:<br>**phylum:** ↔ Firmicutes<br>**genus:** ↑ *Veillonella, Streptocuccus*<br>**species:** ↓ *F.prausnitzii,*↑ *Enterocuccus facecalis*	After RYGB:<br>**phylum:** ↔ Bacteroidetes<br>**genus:** ↑ *Alistipes*	After RYGB:<br>**phylum:** ↔ Actinobacteria<br>**species:** ↑ *Bifidobacterium dentium*	After RYGB:<br>**phylum:** ↑ Proteobacteria<br>**species:** ↑ *E. coli, Klebsiella pneumoniae*	After RYGB:<br>**phylum:** ↔ Verrucomicrobia<br>**species:** ↑ *Akkermansia muciniphila*	After RYGB:<br>**phylum:** ↑ Fusobacteria<br>**species:** ↑ *Fusobacterium nucleatum*
Palmisano *et al.*, 2019	Chao1<br>RYGB: ↔<br>LSG: ↔	Shannon, Simpson<br>RYGB: ↔<br>LSG: ↔	Dissimilarity	After RYGB and LSG:<br>**phylum:** ↔ Firmicutes	After RYGB and LSG:<br>**phylum:** ↔ Bacteroidetes	After RYGB and LSG:<br>**phylum:** ↔ Actinobacteria	After RYGB:<br>**phylum:** ↑ Proteobacteria<br>**class:** ↑ Gammaproteobacteria<br>**species:** ↑ *Yokenella regensburgei*<br>After LSG:<br>**phylum:** ↔ Proteobacteria	After RYGB and LSG:<br>**phylum:** ↔ Verrucomicrobia<br>After RYGB:<br>**species:** ↑ *Akkermansia muciniphila*	N.R.
Patrone *et al.*, 2016	Choa1<br>BIB: ↓	Shannon, Simpson<br>BIB: ↓	Dissimilarity	After BIB:<br>**phylum:** ↔ Firmicutes<br>**family:** ↓ Lactnospiracaea, Clostridiaceae, Ruminococcaceae, Eubacteriaceae<br>**genus:** ↑ *Lactobacillus, Magasphera, Acidaminococcus*	After BIB:<br>**phylum:** ↔ Bacteroidetes	After BIB:<br>**phylum:** ↔ Actinobacteria<br>**family:** ↓ Corio-bacteriaceae	After BIB:<br>**phylum:** ↑ Proteobacteria<br>**family:** ↑ Enterobacteriaceae	After BIB:<br>**phylum:** ↔ Verrucomicrobia	N.R.

Study	Alpha-diversity: richness	Alpha-diversity: biodiveristy	Community Structure	Firmicutes	Bacteroidetes	Actinobacteria	Proteobacteria	Verrucomicrobia	Other
Sanmiguel *et al.*, 2017	LSG: ↔	LSG: ↔	Dissimilarity	After LSG:<br>**phylum:** ↓ Firmicutes<br>**genus:** ↑ *Bulleidia*	After LSG:<br>**phylum:** ↔ Bacteroidetes	After LSG:<br>**phylum:** ↔ Actinobacteria<br>**family:** ↓ Bifidobacteriaceae<br>**genus:** ↑ *Atopobium*	After LSG:<br>**phylum:** ↔ Proteobacteria	After LSG:<br>**phylum:** ↔ Verrucomicrobia	After LSG:<br>**genus:** ↑ *Fusobacterium*
Wang *et al.*, 2019	Chao1<br>LSG: ↑<br>RYGB: ↑	Shannon<br>LSG: ↑<br>RYGB: ↑	Dissimilarity	After RYGB and LSG:<br>**phylum:** ↔ Firmicutes<br>**family:** ↑ Streptococcaceae<br>**genus:** ↑ *Steptoccus*<br>**species:** ↑ *S. Salivarius, S. Thermophilus*<br>After RYGB:<br>**genus:** ↓ *Faecalibacterium*	After RYGB and LSG:<br>**phylum:** ↔ Bacteroidetes<br>After LSG:<br>**family:** ↑ Rikenellaceae, Porphyromonadaceae<br>**genus:** ↑ *Alistipes*<br>**species:** ↑ *Alistipes Finegoldii*	After RYGB and LSG:<br>**phylum:** ↔ Actinobacteria	After RYGB and LSG:<br>**phylum:** ↔ Proteobacteria	After RYGB and LSG:<br>**phylum:** ↔ Verrucomicrobia	After RYGB and LSG:<br>**phylum:** ↔ Fusobacteria

AGB: Adjustable gastric banding; BIB: Bilio-intestinal bypass; ctrl: control group; DJB: Duodenal-jejunal bypass; HWC: Healthy weight controls; LSG: Laparoscopic sleeve gastrectomy; Not reported; RYGB: Roux-en-Y gastric bypass

Table S4b: Subgroup 2 – BS to sham operation comparisons in other vertebrates

Study	Alpha-diversity: richness	Alpha-diversity: biodiversity	Community Structure	Firmicutes	Bacteroidetes	Actinobacteria	Proteobacteria	Verrucomicrobia	Other
Alvarez *et al.*, 2018	Chao1, ACE<br>LSG: ↔	Shannon<br>LSG: ↔	N.R.	After LSG compared to sham:<br>**phylum:** ↔ Firmicutes	After LSG compared to sham:<br>**phylum:** ↔ Bacteroidetes	After LSG2 compared to LSG1 and sham<br>**phylum:**<br>↑ Actinobacteria<br>After LSG1 compared to sham:<br>**phylum:**<br>↔ Actinobacteria	After LSG compared to sham:<br>**phylum:** ↔ Proteobacteria	After LSG compared to sham:<br>**phylum:** ↔ Verrucomicrobia	After LSG compared to sham:<br>**phylum:** ↔ Fusobacteria
Basso *et al.*, 2016	Chao<br>GG: ↔	Shannon<br>GG: ↑	Dissimilarity	After GG compared to sham:<br>**phylum:** ↔ Firmicutes<br>**class:** ↑ Bacilli, Erysipelothrichia, ↓ Clostridia<br>**genus:** ↑ *Lactobacillus,* ↓ *Ruminococcus,* ↔ *Roseburia*	After GG compared to sham:<br>**phylum:** ↔ Bacteroidetes<br>**genus:** ↔ *Bacteroides, Prevotella*	After GG compared to sham:<br>**phylum:** ↔ Actinobacteria<br>**class:** ↑ Actinobacteria<br>**genus:** ↑ *Collinsella*	After GG compared to sham:<br>**phylum:** ↔ Proteobacteria<br>**class:** ↑ Gammaproteobacteria	After GG compared to sham:<br>**phylum:** ↔ Verrucomicrobia	N.R.
Cummings *et al.*, 2013	N.R.	N.R.	N.R.	After IT compared to sham:<br>**phylum:** ↔ Firmicutes<br>**genus**: ↔ *Ruminococcus, Lactobacillus, Roseburia, Oscillibacter*	After IT compared to sham:<br>**phylum:** ↔ Bacteroidetes<br>**genus:** ↔ *Bacteroides, Alistipes*	After IT compared to sham:<br>**phylum:** ↔ Actinobacteria	After IT compared to sham:<br>**phylum:** ↔ Proteobacteria<br>**class:** ↑ Gammaproteobacteria<br>**genus:** ↑ *Escherichia*	After IT compared to sham:<br>**phylum:** ↔ Verrucomicrobia	N.R.
Duboc *et al.*, 2017	Chao1<br>RYGB, LSG: ↔	Shannon, Simpson<br>RYGB, LSG: ↔	Dissimilarity	After RYGB and LSG compared to sham:<br>**phylum:** ↔ Firmicutes<br>**species:** ↓ *Ruminoccocus*<br>After LSG compared to RYGB:<br>**genus:** ↓ *Clostridium*	After RYGB and LSG compared to sham:<br>**phylum:** ↔ Bacteroidetes	After RYGB and LSG compared to sham:<br>**phylum:** ↔ Actinobacteria	After LSG compared to RYGB and sham:<br>**phylum:** ↔ Proteobacteria<br>**genus:** ↑ *Enterobacteriaceae*	After RYGB and LSG compared to sham:<br>**phylum:** ↔ Verrucomicrobia	N.R.
Guo *et al.*, 2016	Chao1, ACE<br>RYGB, LSG: ↔	Shannon<br>RYGB, LSG: ↑	Dissimilarity	After RYGB compared to sham:<br>**phylum:** ↑ Firmicutes<br>After LSG compared to sham:<br>**phylum:** ↓ Firmicutes<br>**class:** ↓ Bacilli<br>After RYGB and LSG compared to sham:<br>**class:** ↑ Clostridia	After LSG compared to sham:<br>**phylum:** ↑ Bacteroidetes<br>**class:** ↑ Bacteroidia<br>After RYGB compared to sham:<br>**phylum:** ↔ Bacteroidetes	After LSG compared to sham:<br>**phylum:** ↑ Actinobacteria<br>After RYGB compared to sham:<br>**phylum:** ↔ Actinobacteria	After RYGB compared to sham:<br>**phylum:** ↑ Proteobacteria<br>**class:** ↑ Betaproteobacteria, gammaproteobacteria<br>After LSG compared to sham:<br>**phylum:** ↔ Proteobacteria	After RYGB and LSG compared to sham:<br>**phylum:** ↔ Verrucomicrobia	After RYGB compared to sham:<br>**phylum:** ↑ Fusobacteria
Huang *et al.*, 2014	N.R.	N.R.	N.R.	After LSG compared to sham:<br>**phylum:** ↔ Firmicutes	After LSG compared to sham:<br>**phylum:** ↔ Bacteroidetes	After LSG compared to sham:<br>**phylum:** ↔ Actinobacteria	After LSG compared to sham:<br>**phylum:** ↔ Proteobacteria	After LSG compared to sham:<br>**phylum:** ↔ Verrucomicrobia	N.R.
Huh *et al.*, 2019	Chao1<br>RYGB, LSG: ↑	N.R.	Dissimilarity	After RYGB and LSG compared to sham:<br>**phylum:** ↓ Firmicutes	After RYGB and LSG compared to sham:<br>**phylum:** ↔ Bacteroidetes	After RYGB and LSG compared to sham:<br>**phylum:** ↔ Actinobacteria	After RYGB and LSG compared to sham:<br>**phylum:** ↑ Proteobacteria	After RYGB and LSG compared to sham:<br>**phylum:** ↔ Verrucomicrobia	N.R.
Jahansouz *et al.*, 2017	ACE<br>LSG: ↔	Shannon<br>LSG: ↔	Similarity	After LSG compared to sham (individually and cohoused):<br>**phylum:** ↓ Firmicutes	After LSG compared to sham (individually and cohoused):<br>**phylum:** ↑ Bacteroidetes	After LSG compared to sham (individually housed):<br>**phylum:** ↔ Actinobacteria<br>After LSG compared to sham (cohoused):<br>**phylum:** ↓ Actinobacteria	After LSG compared to sham (individually and cohoused):<br>**phylum:** ↔ Proteobacteria	After LSG compared to sham (individually and cohoused):<br>**phylum:** ↔ Verrucomicrobia	N.R.

Study	Alpha-diversity: richness	Alpha-diversity: biodiversity	Community Structure	Firmicutes	Bacteroidetes	Actinobacteria	Proteobacteria	Verrucomicrobia	Other
Jiang *et al.*, 2016	N.R.	DJB: ↓	Dissimilarity	After DJB compared to sham: **phylum:** ↑ Firmicutes	After DJB compared to sham: **phylum:** ↓ Bacteroidetes	After DJB compared to sham: **phylum:** ↓ Actinobacteria	After DJB compared to sham: **phylum:** ↓ Proteobacteria	After DJB compared to sham: **phylum:** ↑ Verrucomicrobia **family:** ↑ Verrucomicrobiaceae **genus:** ↑ *Akkermansia*	N.R.
Kashihara *et al.*, 2015	N.R.	N.R.	N.R.	After DJB compared to sham: **phylum:** ↔ Firmicutes **class:** ↔ Bacilli, Clostridia	After DJB compared to sham: **phylum:** ↔ Bacteroidetes **class:** ↓ Bacteroidia	After DJB compared to sham: **phylum:** ↔ Actinobacteria	After DJB compared to sham: **phylum:** ↔ Proteobacteria **class:** ↑ Gammaproteobacteria	After DJB compared to sham: **phylum:** ↔ Verrucomicrobia	N.R.
Kim *et al.*, 2017	N.R.	N.R.	Similarity	After DES compared to sham: **phylum:** ↔ Firmicutes **class:** ↑ Erysipelotrichi **genus:** ↑ *Enterococcus, Allobaculu,* ↓ *Lactobacillus, Streptokokken*	After DES compared to sham: **phylum:** ↓ Bacteroidetes **class:** ↓ Bacteroidia **genus:** ↓ *Prevotella,* ↑ *Parabacteroides*	After DES compared to sham: **phylum:** ↔ Actinobacteria **genus:** ↑ *Bifidobacterium,* ↓ *Rothia*	After DES compared to sham: **phylum:** ↔ Proteobacteria	After DES compared to sham: **phylum:** ↑ Verrucomicrobia **species:** ↑ *Akkermansia muciniphila*	After DES compared to sham: **phylum:** ↑ Mollicutes
Li J.V. *et al.*, 2011	N.R.	N.R.	N.R.	After RYGB compared to sham: **phylum:** ↑ Firmicutes **class:** ↔ Bacilli, ↓ Clostridia	After RYGB compared to sham: **phylum:** ↔ Bacteroidetes	After RYGB compared to sham: **phylum:** ↔ Actinobacteria	After RYGB compared to sham: **phylum:** ↑ Proteobacteria **class:** ↑ Gammproteobacteria	After RYGB compared to sham: **phylum:** ↔ Verrucomicrobia	N.R.
Li S. *et al.*, 2017	N.R.	N.R.	N.R.	After DJB and LSG compared to sham: **family:** ↑ Lactobacillales	N.R.	N.R.	N.R.	N.R.	N.R.
Liou *et al.*, 2013	N.R.	N.R.	Dissimilarity	After RYGB compared to sham: **phylum:** ↓ Firmicutes **order:** ↑ Clostridiales **genus:** ↑ *Clostridium,* ↓ *Lactobacillus*	After RYGB compared to sham: **phylum:** ↑ Bacteroidetes **order:** ↔ Bacteroidales **genus:** ↑ *Alistipes*	After RYGB compared to sham: **phylum:** ↔ Actinobacteria	After RYGB compared to sham: **phylum:** ↑ Proteobacteria **order:** ↑ Enterobacteriales	After RYGB compared to sham: **phylum:** ↑ Verrucomicrobia **order:** ↑ Verrucomicrobiales **genus:** ↑ *Akkermansia*	N.R.
Liu *et al.*, 2018	N.R.	Shannon RYGB: ↔	Dissimilarity	After RYGB compared to sham: **phylum:** ↓ Firmicutes **order:** ↓ Clostridiales, ↑ Lactobacillales	After RYGB compared to sham: **phylum:** ↑ Bacteroidetes	After RYGB compared to sham: **phylum:** ↔ Actinobacteria	After RYGB compared to sham: **phylum:** ↔ Proteobacteria **order:** ↑ Enterobacteriales, Burkholderiales	After RYGB compared to sham: **phylum:** ↔ Verrucomicrobia **order:** ↑ Verrucomicrobiales	After RYGB compared to sham: **phylum:** ↑ Fusobacteria
Miyachi *et al.*, 2017	N.R.	N.R.	N.R.	After DJB comapred to sham: **phylum:** ↔ Firmicutes After B-DJB compared to J-DJB and sham: **genus:** ↓ *Clostridium, Turicibacter*	After DJB compared to sham: **phylum:** ↔ Bacteroidetes After B-DJB compared to J-DJB and sham: **genus:** ↓ *Bacteroides*	After DJB compared to sham: **phylum:** ↔ Actinobacteria After B-DJB compared to J-DJB and sham: **genus:** ↑ *Bifidobacterium, Olsenella*	After DJB compared to sham: **phylum:** ↔ Proteobacteria	After DJB compared to sham: **phylum:** ↔ Verrucomicrobia	N.R.
Mukorako *et al.*, 2019	Chao1 BPD/DS, LSG: ↔ DS: ↓	Shannon BPD/DS, DS: ↓ LSG: ↔	Dissimilarity	After BDP/DS, DS and LSG compared to sham: **phylum:** ↔ Firmicutes After BDP/DS and DS compared to sham: **class:** ↓ Clostridiales **family:** ↓ Peptostreptococcaceae, Clostridiaceae	After BDP/DS, DS and LSG compared to sham: **phylum:** ↔ Bacteroidetes After BDP/DS and DS compared to sham: **order:** ↓ Bacteroidales	After BDP/DS, DS and LSG compared to sham: **phylum:** ↔ Actinobacteria After BDP/DS and DS compared to sham: **order:** ↑ Bifidobactiales **genus:** ↑ *Bifidobacterium*	After BDP/DS, DS and LSG compared to sham: **phylum:** ↔ Proteobacteria	After BDP/DS, DS and LSG compared to sham: **phylum:** ↔ Verrucomicrobia	N.R.

Study	Alpha-diversity: richness	Alpha-diversity: biodiversity	Community Structure	Firmicutes	Bacteroidetes	Actinobacteria	Proteobacteria	Verrucomicrobia	Other
Shao *et al.*, 2017	N.R.	Shannon RYGB: ↓ compared to LSG and Sham	LSG: Similarity RYGB: Dissimilarity	After RYGB and LSG compared to sham: **phylum:** ↔ Firmicutes	After RYGB and LSG compared to sham: **phylum:** ↔ Bacteroidetes	After RYGB and LSG compared to sham: **phylum:** ↔ Actinobacteria	After RYGB compared to sham: **phylum:** ↑ Proteobacteria **class:** ↑ Gammaproteobacteria After LSG compared to sham: **phylum:** ↔ Proteobacteria	After RYGB and LSG compared to sham: **phylum:** ↔ Verrucomicrobia	N.R.
Shao *et al.*, 2018	Chao1 LSG: ↑	Shannon LSG: ↔	Dissimilarity	After LSG compared to sham: **phylum:** ↓ Firmicutes **class:** ↓ Erysipelotrichia, Bacilli	After LSG compared to sham: **phylum:** ↑ Bacteroidetes	After LSG compared to sham: **phylum:** ↔ Actinobacteria	After LSG compared to sham: **phylum:** ↔ Proteobacteria	After LSG compared to sham: **phylum:** ↑ Verrucomicrobia	N.R.
Wang *et al.*, 2019	N.R.	N.R.	N.R.	After RYGB compared to sham: **phylum:** ↓ Firmicutes **order:** ↓ Lactobacillales, ↔ Erysipelotrichales **family:** ↓ Clostridiaceae	After RYGB compared to sham: **phylum:** ↔ Bacteroidetes	After RYGB compared to sham: **phylum:** ↔ Actinobacteria	After RYGB compared to sham: **phylum:** ↑ Proteobacteria **order:** ↑ Enterobacteriales	After RYGB compared to sham: **phylum:** ↔ Verrucomicrobia	N.R.
Zhang X. *et al.*, 2016	Chao1, ACE DJB: ↔	N.R.	N.R.	After DJB compared to sham: **phylum:** ↑ Firmicutes	After DJB compared to sham: **phylum:** ↓ Bacteroidetes	After DJB compared to sham: **phylum:** ↔ Actinobacteria	After DJB compared to sham: **phylum:** ↑ Proteobacteria	After DJB compared to sham: **phylum:** ↔ Verrucomicrobia	N.R.

BPD/DS: Bilopancreal diversion with duodenal switch; ctrl: Control group; DES: Duodenal endoluminal barrier sleeve; DJB: Duodenal-jejunal bypass (B-DJB, J-DJB); DS: Duodenal switch; GG: Glandular gastrectomy; IT: Ileal Interposition; LSG: Laparoscopic sleeve gastrectomy; N.R.: Not reported; RYGB: Roux-en-Y gastric bypass

Table S5 – OHAT: Risk of Bias

	SB			CB	PB		A/EB	DB			SRB	OB	
**Author (year)**	**1**	**2**	**3**	**4**	**5**	**6**	**7**	**8**	**9**		**10**	**11**	**12**
Group 1: Bariatric surgery and microbiota in humans													
Aron-Wisnewsky et al. (2018)	NA	NA	_	+	NA	NA	_	+	+	NR	++	+	_
Bjorneklett et al. (1981)	NR	NR	NA	NA	NA	NR	NR	+	_	NR	+	+	NR
Campisciano et al. (2017)	_	NR	NA	NA	NA	NR	NR	+	++	NR	+	+	NR
Campisciano et al. (2018)	_	NR	NA	NA	NA	NR	NR	+	++	NR	+	+	NR
Chen et al. (2017)	NA	NA	NA	NR	NA	NA	NR	++	++	NR	++	+	++
Cortez et al. (2018)	NR	NR	NA	NA	NA	NR	_	_	++	NR	+	+	++
Damms-Machado et al. (2015)	NA	NA	+	NR	NA	NA	_	++	+	NR	+	+	++
Federico et al. (2016)	NA	NA	+	NR	NA	NA	+	++	++	NR	++	+	+
Fouladi et al. (2019)	NA	NA	+	+	NA	NA	++	+	+	NR	+	+	NR
Furet et al. (2010)	NA	NA	NR	+	NA	NA	_	+	++	NR	++	++	+
Graessler et al. (2013)	NA	NA	NA	++	NA	NA	++	+	++	NR	++	++	+
Gutierrez-Repiso et al. (2019)	NA	NA	+	NR	NA	NA	NR	++	+	NR	++	+	_
Ilhan et al. (2017)	NA	NA	+	+	NA	NA	++	+	+	NR	++	++	++
Kellerer et al. (2019)	NA	NA	+	+	+	NA	++	++	++	NR	++	+	+
Kong et al. (2013)	NA	NA	NA	NR	NA	NA	NR	+	++	NR	++	+	+
Lee et al. (2019)	++	++	NA	NA	NA	+	+	NR	+	+	+	+	++
Lin et al. (2019)	NA	NA	+	_	NA	NA	NR	+	++	NR	+	+	++
Liu et al. (2017)	NA	NA	++	+	NA	NA	++	++	++	_ _	++	++	+
Medina et al. (2017)	NA	NA	+	_	NA	NA	++	++	++	NR	++	+	+
Murphy et al. (2017)	+	+	NA	NA	NA	+	_	+	+	+	++	+	+
Paganlli et al. (2019)	NA	NA	++	++	NA	NA	++	++	+	++	NR	++	++
Pajecki et al. (2019)	NA	NA	NA	NR	NA	NA	+	++	++	NR	+	+	_
Palleja et al. (2016)	NA	NA	NA	NR	NA	NA	NR	+	++	NR	++	+	+
Palmisano et al. (2019)	NA	NA	+	+	NA	NA	++	+	++	NR	++	++	+
Patrone et al. (2016)	NA	NA	NA	++	NA	NA	++	++	++	NR	++	+	NR
Rosina et al. (1993)	NA	NA	NA	+	NA	NA	+	++	+	NR	++	+	NR
Sanmiguel et al. (2017)	NA	NA	NA	+	NA	NA	_ _	++	++	NR	++	+	NR
Tremaroli et al. (2015)	NA	NA	++	NR	NA	NA	++	++	++	+	++	+	+
Wang et al. (2019)	NA	NA	_	++	NA	NA	_ _	+	++	NR	+	+	NR
Zhang et al. (2009)	NA	NA	NR	+	NA	NA	++	++	++	NR	++	+	NR
Group 2: Bariatric surgery and microbiota in other vertebrates													
Alvarez et al. (2018)	+	NA			++	NA	++	+	+	NA	++	++	NA
Basso et al.(2016)	+	NA			++	NA	_	+	+	NA	+	+	NA
Bastos et al. (2018)	+	NA			++	NA	+	+	++	NA	+	+	NA
Cummings et al. (2013)	_ _	NA			++	NA	_	+	++	NA	++	+	NA
Duboc et al. (2017)	_ _	NA			++	NA	NA	+	+	NA	+	_ _	NA
Guo et al. (2016)	+	NA			+	NA	_	+	+	NA	+	+	NA
Huang et al. (2014)	+	NA			+	NA	+	+	+	NA	+	_	NA
Huh et al.(2019)	++	NA			++	NA	+	++	+	NA	+	+	NA
Jahansouz et al. (2017)	+	NA			_	NA	+	+	+	NA	+	+	NA
Jiang et al. (2016)	_	NA			+	NA	_	+	_	NA	_	++	NA
Kashihara et al. (2015)	+	NA			+	NA	_ _	_ _	_ _	NA	_ _	_ _	NA
Kim et al. (2017)	_ _	NA			+	NA	_	+	+	NA	+	+	NA
Li J.V. et al. (2011)	_	NA			+	NA	NA	+	+	NA	+	+	NA
Li S. et al.(2017)	_	NA			+	NA	+	+	+	NA	+	+	NA
Liou et al.(2013)	_	NA			+	NA	_	_	+	NA	+	_	NA
Liu et al.(2018)	_	NA			+	NA	_	_	+	NA	+	_	NA
Miyachi et al.(2017)	+	NA			+	NA	_	+	+	NA	+	+	NA
Mukorako et al.(2019)	+	NA			++	NA	+	++	+	NA	+	++	NA
Osto et al.(2013)	+	NA			+	NA	NA	+	+	NA	+	+	NA
Schippers et al.(1996)	NA	NA			+	NA	++	++	+	NA	+	+	++
Shao et al.(2017)	+	NA			+	NA	+	+	+	NA	+	_ _	NA
Shao et al.(2018)	+	NA			+	NA	_	_	+	NA	+	+	NA

Wang et al. (2019)	++	NA			++	NA	+	++	+	NA	+	++	NA
Yang et al.(2015)	+	NA			+	NA	NA	NA	+	NA	+	+	NA
Zhang X. et al. (2015)	_	NA			+	NA	+	+	+	NA	+	+	NA
Group 3: Bariatric surgery and probiotics													
Chen et al. (2016)	+	++	NA	NA	NA	+	+	+	++	++	++	+	+
Kazzi et al. (2018)	NR	NR	NA	NA	NA	_ _	_	+	++	_ _	_ _	++	++
Sherf-Dagan et al. (2018)	++	++	NA	NA	NA	++	+	++	++	++	++	+	+
Woodard et al. (2009)	++	++	NA	NA	NA	_ _	+	+	+	_ _	_ _	+	+

Legend: definitely low: “++”; probably low: “+”; probably high: “-”; definitely high risk of bias: “--”; “NA”: not applicable; “NR”: not reported.
